# Loss of serine/threonine protein phosphatase 6 severely impairs sexual stage development in malaria parasite *Plasmodium berghei*

**DOI:** 10.1371/journal.ppat.1013318

**Published:** 2025-07-07

**Authors:** Yonghui Feng, Wenyan Gao, Chengqi Wang, Shuangrui Shi, Dan Zhou, Lin Sun, Liying Zhu, Liwang Cui, Yaming Cao, Xiaotong Zhu

**Affiliations:** 1 Department of Immunology, College of Basic Medical Sciences, China Medical University, Shenyang, Liaoning, China; 2 Department of Laboratory Medicine, The First Hospital of China Medical University, Shenyang, Liaoning, China; 3 Department of Obstetrics, The First Hospital of China Medical University, Shenyang, Liaoning, China; 4 Center for Global Health and Infectious Diseases Research, College of Public Health, University of South Florida, Tampa, Florida, United States of America; 5 Department of Internal Medicine, Morsani College of Medicine, University of South Florida, Tampa, Florida, United States of America; Bernhard-Nocht-Institute for Tropical Medicine, GERMANY

## Abstract

Protein phosphorylation plays a critical role during the development of malaria parasites. Here, we performed a functional analysis of the *Plasmodium berghei* Ser/Thr protein phosphatase 6 (PbPP6), which is associated with the plasma membrane of macrogametes and ookinetes. Compared to wild-type *P. berghei*, the genetic disruption of *pbpp6* (∆*pbpp6*) resulted in reduced asexual growth of the parasites and prolonged survival of infected mice. The ∆*pbpp6* parasites showed impaired gametogenesis, particularly affecting male gametogenesis, which substantially decreased both ookinete formation and mosquito transmission. Transcriptomic analysis revealed an over 11-fold downregulation of *nek3*, a regulator of MAPK2 within the PKG-Ca^2^⁺ signaling cascade, foreshadowing pathway dysregulation that was further evidenced by significantly diminished intracellular cGMP levels, decreased cytosolic Ca^2^⁺ mobilization, and reduced DNA replication in activated Δ*pbpp6* gametocytes. Phosphoproteomic analysis detected increased phosphorylation at the Ser508 site of guanylyl cyclase alpha (GCα), indicating that PbPP6 regulates cGMP-PKG-Ca^2+^ signaling through modulation of GCα activity during gametogenesis. Additionally, we observed altered expression of messenger ribonucleoproteins in the Δ*pbpp6* parasites, which may affect the translational repression of stored mRNAs in female gametocytes and impact post-fertilization development in mosquitoes. Collectively, this study highlights the potential of targeting PP6 to disrupt malaria transmission.

## Author summary

Malaria transmission begins when *Plasmodium* gametocytes, ingested by a female *Anopheles* mosquito, activate and differentiate into gametes in the midgut. Through genetic and multi-omics analyses, we identified the protein phosphatase PbPP6 as a key regulator of the cGMP-PKG-Ca^2^⁺ signaling cascade, specifically via the dephosphorylation of GCα. Disruption of the *pbpp6* gene impaired male gametogenesis and affected post-fertilization development, likely due to dysregulated mRNP-mediated translational repression. Our findings demonstrate that genetic disruption of PbPP6 drastically reduced parasite transmission, highlighting protein phosphatases as potential targets for blocking malaria transmission.

## Introduction

The World Health Organization has launched the “Global Technical Strategy for Malaria 2016 – 2030” to accelerate malaria elimination [1]. However, malaria chemotherapy, one of the technical pillars of malaria control and elimination, is challenged by the emergence of multidrug-resistant parasites. Of particular concern is the emergence and spread of *Plasmodium falciparum* parasites resistant to artemisinins and their partner drugs in Southeast Asia [2]. Thus, developing new antimalarial drugs is a high priority. In recognition of the significance of interrupting malaria transmission, it is desired that antimalarial candidates not only clear asexual parasitemia but also block parasite transmission. For drug discovery, a better understanding of parasite biology is critical for identifying novel drug targets [3].

Nearly half of the total proteins from humans, mouse and yeast are subject to phosphorylation, the most frequent post-translational modification [4]. Reversible phosphorylation, mediated by the opposing actions of kinases and phosphatases, also plays essential roles in malaria parasites, regulating cell division, propagation, stage conversion, and pathogenesis [5,6]. The *P. falciparum* genome encodes approximately 85 putative protein kinases and 30 protein phosphatases [7–9]. Compared with protein kinases, protein phosphatases in malaria parasites have received much less attention and deserve more detailed investigations, given their recognized potentials as therapeutic targets. The *Plasmodium* phosphatome is highly conserved among species [9]; thus, studies using the genetically more amenable rodent malaria parasite *Plasmodium berghei* may yield information that is directly relevant to the human malaria parasites. The *Plasmodium* protein phosphatases are divided into five families: 11 phosphoprotein phosphatases (PPPs), 10 metallo-protein phosphatases (PPMs), 4 protein tyrosine phosphatases (PTPs) and a PTP-like phosphatase, and 5 NLI-interacting factor-like phosphatases (NIFs) [8,9]. A systematic functional analysis of the *P. berghei* phosphatome revealed that 16 out of the 30 predicted protein phosphatases are essential for asexual stage development, while six are required for transmission to mosquitoes [9]. Although the study indicated that all major members of the PPP subfamily (PP1–PP8) are essential for asexual erythrocytic development [9], we found that PbPP5 is not required for this process but is crucial for male gamete formation and fertility [10]. Within the PPP subfamily, *P. berghei* protein phosphatase 6 (*pbpp6*, *PbANKA_0412100*) is also expressed in gametocytes and ookinetes [11], suggesting its potential involvement in sexual development. PP6 members are conserved among eukaryotes, and they are involved in intracellular signaling [12–14]. In *Toxoplasma gondii* (*T. gondii*), TgPP6C is essential for the parasite replication and its deletion could significantly attenuate the parasite virulence in mice [15]. In yeast, PP6 plays a non-redundant function in regulating cell cycle progression from G1 to S phase, which cannot be complemented by other closely-related protein phosphatases such as PP2A and PP4 [16,17].

In this study, we performed functional characterization of PbPP6 during the asexual erythrocytic cycle and sexual development of *P. berghei*. We found that PbPP6 was up-regulated during sexual development and located underneath the parasite plasma membrane at the gamete and ookinete stages. Functional analyses showed that *pbpp6* deletion significantly impaired sexual development and infection of mosquito midguts. To understand the underlying mechanisms, we explored the effect of *pbpp6* deletion on transcriptome, proteome and phosphoproteome in gametocytes.

## Results

### Validation of protein phosphatase activity and expression of the PbPP6 protein

PbPP6, encoded by the *PbANKA_041210* gene, is a protein of 308 amino acids (aa) with a predicted molecular mass of 35.8 kDa. The phosphatase domain (PP2Ac, 21–292 aa) contains several identifiable motifs, such as the protein kinase C phosphorylation sites (TKK and SVK) and tyrosine kinase phosphorylation site (KYCTDIFDY) (S1A Fig). The PP2Ac domain shares significant homology to PP6-like proteins from different organisms, with six highly conserved core motifs ‘GDIHG’, ‘GDYVDRG’, ‘GNHE’, ‘HGG’, ‘RG’ and ‘H’ at the proposed catalytic site (S1B Fig). Although the sequence identify between PbPP6 and TgPP6C is only 24% when comparing the full legth protein sequence, the sequence identity of PP2Ac domain between these two protein is of 74% (S1B Fig). Furthermore, PP6 is highly conserved in *Plasmodium* spp., PbPP6 is 95.1% identical to its ortholog PfPP6 in *P. falciparum*. Phylogenetic analysis based on the full-length proteins of the PP6 orthologs revealed that the apicomplexan PP6 proteins are monophyletic (S1C Fig).

**Fig 1:**
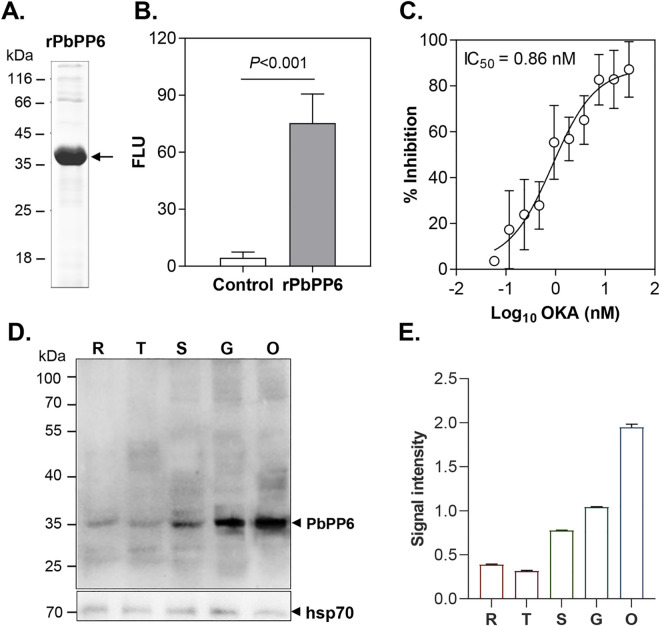
Enzyme activity and drug inhibition assays. A. Recombinant PbPP6 (rPbPP6) was expressed and purified, followed by SDS-PAGE analysis. The arrow indicates the expected ~38 kDa band. B. The phosphatase activity of rPbPP6 and a His-tagged Trx-A protein (control) was measured using a phosphorylated S/T PPase R110 substrate (-FLU, fluorescence light units). C. The dose-dependent inhibition of rPbPP6 by okadaic acid (OKA) was assessed. The error bars represent ±SD (biological triplicates). D. Western blot analysis of PbPP6 expression profile. The endogenous PbPP6 was detected using anti-PbPP6 sera (predicted band: 35.8 kDa). R, Ring; T, Trophozoite; S, Schizont; G, Gametocyte; O, Ookinete. Equal loading of samples was verified using mouse anti-Hsp70 sera. Arrows denote the expected bands. Representative data from three biological replicates are shown. E. The relative protein expression levels of PbPP6 (normalized to Hsp70) were quantified from (D) using ImageJ software.

To confirm the prediction of PbPP6 as a PP2Ac phosphatase, we expressed the full-length PbPP6 in the *Pichia* expression system. With a His tag fused to the C-terminus of the protein, the recombinant PbPP6 protein (rPbPP6) was purified by using Ni-NTA chromatography. Analysis of rPbPP6 on an SDS-PAGE gel revealed a protein band of ~38 kDa, consistent with its predicted molecular weight (Fig 1A). Using the phosphorylated Ser/Thr PPase R110 as a substrate, the rPbPP6 exhibited obvious phosphatase activity, in stark contrast to the control recombinant His-tag protein (*P *< 0.001, Fig 1B). rPbPP6 exhibited high sensitivity to okadaic acid, with an IC_50_ (the concentration that inhibits 50% of enzymatic activity) as low as 0.86 nM (Fig 1C), which is lower than that of rPbPP5 [10].

To determine the expression and subcellular localization of PbPP6, we generated polyclonal antisera against PbPP6 by immunizing mice with rPbPP6. Since transcriptomic analysis showed *pbpp6* expression during the life cycle of malaria parasites [18], we purified different parasite stages for analysis (S2 Fig). Western blots with protein extracts from purified rings, trophozoites, schizonts, gametocytes, and ookinetes using the anti-rPbPP6 antisera detected PbPP6 expression as a 36 kDa protein band in all stages analyzed (Fig 1D). During asexual intraerythrocytic development, the PbPP6 expression level was relatively low in the ring and trophozoite stages, but it increased by approximately 2-fold during the schizont stage. In contrast, PbPP6 was predominantly expressed in the gametocyte and ookinete stages (Fig 1D and 1E).

### Localization and association of PbPP6 with the peripheral membrane in sexual stages

**Fig 2:**
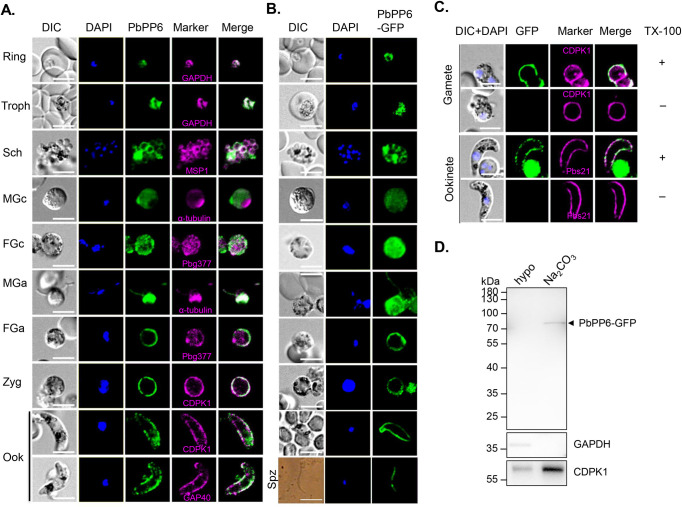
Subcellular localization of endogenous PbPP6 and PbPP6-GFP fusion protein. **A-B.** IFA analysis for location of endogenous PbPP6 (**A**) and PbPP6-GFP fusion protein (**B**) at various stages of *Plasmodium berghei*. Troph, Trophozoite; Sch, Schizont; MGc, Male gametocyte; FGc, Female gametocyte; MGa, Male gamete; FGa, Female gamete; Ook, Ookinete. (A) Endogenous PbPP6 was detected using Alexa Fluor 488 (Green). Phase contrast (DIC), DAPI-stained nuclei (blue), Alexa Fluor 594 channel (magenta; stage-specific markers), and merged images are presented. (B) PbPP6-GFP fluorescence (green) is shown alongside DIC and DAPI (blue). Scale bar = 5 μm. Note that different images were taken by non-consistence exposure time, which were not meant for quantitative comparison. C. IFA analysis assessing PbPP6 localization in gametes and ookinetes following fixation with (+) or without (-) treatment using 0.1% Triton X-100 (TX-100). Fixed Parasites were treated with (+)/(-) TX-100 before being stained with anti-GFP (detected via Alexa Fluor 488; green). After staining, all samples were universally permeabilized with TX-100 and stained with co-localization markers (CDPK1 or Pbs21; Alexa Fluor 594; magenta). The DAPI channel (blue) was used to visualize nucleus. Alexa488 and Alexa594 images were merged to show concordance. Scale bar = 5 μm. D. Subcellular fractionation of PbPP6-GFP. Parasite lysates were separated into soluble cytosolic (obtained through hypotonic lysis; Hypo) and peripheral membrane (sodium carbonate-extracted; Na₂CO₃) fractions. PbPP6-GFP (black arrow) was detected by immunoblotting using an anti-GFP antibody. Anti-GAPDH and anti-CDPK1 antibodies were used as controls for cytosolic and membrane-associated proteins, respectively.

To determine the subcellular locations of the PbPP6 protein, we performed an indirect immunofluorescence assay (IFA) in wild-type (WT) *P. berghei* ANKA strain using antisera against rPbPP6 and antibodies against proteins associated with known cellular compartments. In the ring and trophozoite stages, PbPP6 fluorescence showed a diffused pattern with one or a few brighter puncta, largely overlapping with the cytoplasmic marker *P. berghei* glyceraldehyde-3-phosphate dehydrogenase (GAPDH) (Fig 2A). In schizonts, whereas the PbPP6 showed substantial co-localization with the merozoite surface protein 1 (MSP1), much of the fluorescence also emanated from the cell periphery suggesting a plasmalemma location pattern (Fig 2A). In both male and female gametocytes, the fluorescence was distributed throughout the cytoplasm with many prominent puncta. In microgametes, the fluorescence signal was associated with both the flagella and the residual body, co-localized with the α-tubulin II protein (Fig 2A). In macrogametes, zygotes and ookinetes, however, the PbPP6 signal pattern resembled both the plasma membrane (CDPK1) and the IMC marker (GAP40).

To perform cross-validation, we engineered a transgenic line of *P. berghei* (designated PbPP6-GFP) in which the endogenous PbPP6 protein was tagged at the C-terminally with 2 × FKBP, followed by a green fluorescent protein (GFP) (S3A Fig). The correct integration of the tags at the *pbpp6* locus was confirmed by diagnostic PCR (S3B Fig). Western blot analyses of protein extracts from mixed asexual stage parasites, using an anti-GFP monoclonal antibody (mAb), revealed a protein band of approximately 90 kDa, which aligns with the expected size of the PbPP6-GFP fusion protein (S3C Fig). The PbPP6-GFP parasites did not show any noticeable growth defects during the blood stage (S3D Fig) and showed no significant difference in virulence (S3E Fig), nor did they interfere with transmission through *Anopheles stephensi* mosquitoes (S3F and S3G Fig). IFA of the PbPP6-GFP line, using anti-GFP antibodies, revealed similar location patterns of PbPP6-GFP in various developmental stages as PbPP6 in WT parasites detected with the anti-rPbPP6 antisera (Fig 2B). Additionally, sporozoites dissected from the salivary glands of infected mosquitoes at 21 days post infection (dpi) displayed cytosolic GFP fluorescence (Fig 2B).

To further examine the association of PbPP6 with the plasma membrane in macrogametes and ookinetes, we conducted co-localization studies with and without treatment using Triton X-100 for membrane permeabilization. PbPP6-GFP was only detected following Triton X-100 treatment, suggesting that PbPP6 was located beneath the parasite plasma membrane of the parasite (Fig 2C). To determine whether PbPP6-GFP was directly associated with the cytoplasmic membrane in ookinetes, purified ookinetes were fractioned into the cytosolic and membrane-associated fractions using a hypotonic reagent and sodium carbonate, respectively. The proteins in both fractions were then analyzed using SDS-PAGE and western blotting. The results showed the presence of PbPP6-GFP only in the carbonate-soluble fraction (which contains peripheral membrane proteins), suggesting that PbPP6 is a membrane-associated protein in ookinetes (Fig 2D).

### Perturbation of sexual development upon *pbpp6* knockout

Previous functional studies of human and rodent malaria parasites suggested that *pbpp6* gene is possibly essential [9]. To investigate the function of PbPP6, we attempted to knock out the *pbpp6* gene by using both double and single crossover homologous recombination strategies. In both experiments, we were able to obtain parasite clones that had *pbpp6* either completely deleted or partially disrupted (deleting aa 45–308), as verified by genotyping PCR, western blots, and IFA analysis (S4A–C Fig). Two *pbpp6* knockout clones, Δ*pbpp6* K1 (K1) and Δ*pbpp6* K2 (K2), from two independent transfection attempts, were selected for further analysis. To determine whether *pbpp6* deletion affected asexual parasite growth, mice were infected by injection of 10^6^ parasitized red blood cells (RBCs) of the WT *P. berghei*, K1 and K2, respectively. The two Δ*pbpp6* clones showed indistinguishable growth curves, and the initial daily parasitemias were also similar between the Δ*pbpp6* and WT parasites (S4D Fig). However, from 6 dpi onwards, the daily parasitemias were significantly lower in mice infected with the Δ*pbpp6* parasites than in mice infected with the WT parasites (S4D Fig, *P* < 0.01, Student’s *t*-test). Specifically, the mean parasitemias at 6 and 14 dpi in the two Δ*pbpp6* groups reached 7.1% and 41.3%, respectively, compared to 8.1% and 45.8% in the WT group (S4D Fig). Correspondingly, mortality of Δ*pbpp6*-infected mice was also delayed for about two days (S4E Fig, *P* < 0.01, Kaplan–Meier’s survival analysis).

**Fig 3:**
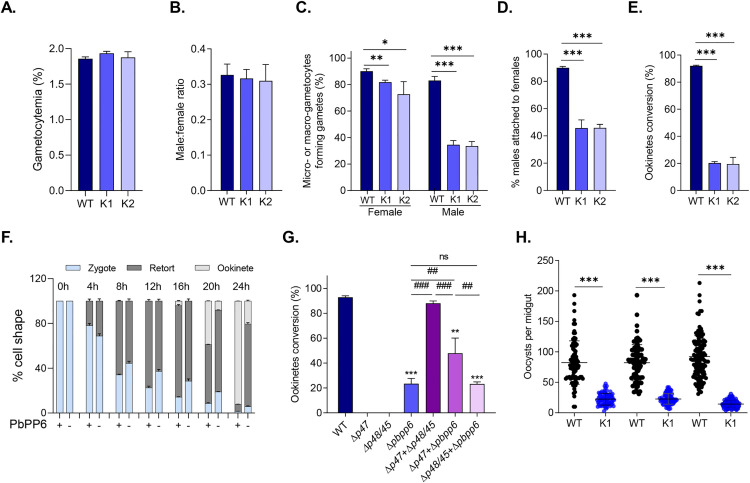
The sexual stage phenotype of *pbpp6* gene knockout clones. A. Gametocytemia in WT and ∆*pbpp6* parasites at day 3 post-infection (p.i.). B. Ratios of male to female gametocytes at day 3 p.i. C. Efficiency of macro- and microgamete formation. D. Interaction between male and female gametes. E. Ookinete conversion rates, measured as the percentage of Pbs21-positive parasites that developed into mature ookinetes, identified through anti-Pbs21 mAb staining. F. Developmental progression of Δ*pbpp6* and WT parasites during ookinete differentiation, with zygotes (cyan), retorts (dark grey), and mature ookinetes (light grey) quantified using fluorescence microscopy (anti-Pbs21 mAb). G. Ookinete conversion rates in genetic crosses between Δ*pbpp6* and female-defective (Δ*p47*) or male-defective (Δ*p48/45*) mutants. H. Oocyst counts per mosquito midgut at day 10 post-infection. For (A-H), data represent mean ± SD from three biological replicates unless otherwise noted. In (F), means were derived from two independent experiments (≥300 parasites scored per time point). Statistical significance versus WT is indicated as follows: * *P* < 0.05, ** *P* < 0.01, *** *P* < 0.001; for intergroup comparisons: n.s. (not significant), ## *P* < 0.01, ### *P* < 0.001. The samples sizes for mosquitoes were as follows: Experiment 1 (n = 85), Experiment 2 (n = 95), Experiment 3 (n = 120).

Given the predominant expression of PbPP6 in gametocytes and ookinetes, we wanted to determine the impact of *pbpp6* deletion on sexual development. We observed similar levels of gametocytemia and sex ratio at 3 dpi in WT and Δ*pbpp6* parasites, when gametocytemia normally peaks, indicating that *pbpp6* knockout did not affect gametocytogenesis (Fig 3A and 3B, Student’s *t* test). However, the gametogenesis efficiencies of both male and female gametocytes were significantly affected. Compared to the WT, the proportion of macrogametocytes forming macrogametes was reduced by 8.3% and 17.4% in the K1 and K2 lines, respectively, whereas the proportion of microgametocytes forming exflagellation centers was reduced by 48.5% and 49.5% in the K1 and K2 lines, respectively (Fig 3C). We also scored the proportion of positive male-female gamete interactions, defined as male-female attachment lasting for more than 3 sec, during a period of 20 min from 10 min after induction for gametogenesis. The results revealed that the proportion of positive male-female interactions was reduced by 44.3% and 44.1% in Δ*pbpp6* K1 and K2, respectively (Fig 3D). We further determined the *in vitro* ookinete conversion rates of the WT and mutant lines. At 24 h of the ookinete culture, 20.3% and 19.5% of the Δ*pbpp6* macrogametocytes were converted into morphologically mature ookinetes in K1 and K2, respectively, significantly lower than the rate (92.1%) in the WT (Fig 3E, *P* < 0.001, Student’s *t* test). We then performed a time-course analysis to determine the step(s) in the zygote-ookinete development affected by the *pbpp6* deletion. Compared with WT, the Δ*pbpp6* K1 parasite began to show a delay in the morphological transformation from 4 h post-fertilization (Fig 3F). At 16 h post-fertilization, 4.0% of WT parasites appeared as mature ookinetes compared to none in the Δ*pbpp6* K1 parasites. At 24 h, the majority (92.2%) of the WT parasites developed into mature ookinetes, which was in sharp contrast to 72.8% of the Δ*pbpp6* K1 parasites remaining in the “retort” stage (Fig 3F). These results indicated that PbPP6 had essential functions in gametogenesis, fertilization, and post-zygotic development of *P. berghei*.

To investigate whether the defects in sexual development upon *pbpp6* deletion were sex-specific, we conducted *in vitro* genetic crossover experiments by mixing equal numbers of mature gametocytes of Δ*pbpp6* K1 with either the male-defective (Δ*p48/45*) or female-defective (Δ*p47*) gametocytes and culturing them for 24 h. Whereas the cross between the Δ*p48/45* or Δ*p47* parasites restored ookinete conversion rate to the level of WT parasites, the cross between Δ*pbpp6* and Δ*p48/45* parasites did not rescue the ookinete conversion defects of the Δ*pbpp6* parasite line (Fig 3G). Although the cross between Δ*pbpp6* and Δ*p47* parasites could partially rescue the phenotype, the ookinete conversion defects is still 40.2% lower than the cross between Δ*p48/45* and Δ*p47* parasites, suggesting that both male and female gametes were impaired in the Δ*pbpp6* lines (Fig 3G).

In mosquito feeding assays, *An. stephensi* mosquitoes feeding on Δ*pbpp6*-infected mice showed a 76.9% reduction in midgut oocyst intensity compared to those feeding on WT *P. berghei*-infected mice (Mann–Whitney *U* test, *P* < 0.001, Fig 3H and Table 1). In addition, the infection prevalence of mosquitoes feeding on Δ*pbpp6*-infected mice (82.8%) was also significantly reduced as compared to mosquitoes feeding on WT*-*infected mice (97.4%) (Fisher’s exact test, *P* < 0.001, Table 1). Likewise, the sporozoite load in the infected salivary glands were also drastically reduced in mosquitoes feeding on Δ*pbpp6*-infected mice (Table 1). To determine sporozoite viability, we purified sporozoites from the mosquito salivary glands at 20 dpi and injected them intravenously into BALB/c mice at 3000 sporozoites/mouse. Under these conditions, the Δ*pbpp6* and WT sporozoites displayed similar efficiencies in infecting mice; all injected mice developed blood-stage infections with similar pre-patent periods and parasitemia levels (Table 1). Together, these results showed that *pbpp6* is essential for sexual stage development in mosquitoes.

**Table 1:** Reduced oocyst intensity and infection prevalence in mosquito fed on Δ*pbpp6*-infected mice and sporogonic development.

	WT			Δ*pbpp6*		
	M1	M2	M3	M1	M2	M3
Mosquitoes infected/Dissected	82/85	91/95	120/120	71/85	76/95	102/120
Prevalence of infection (%)^a^	96.5	95.8	100.0	83.5	80.0	85.0
Mean prevalence (%)			97.4			82.8
Reduction in prevalence (%)^b^						14.6^***^
Oocyst intensity^c^	82.5 ± 3.9	82.2 ± 3.1	92.2 ± 3.2	22.4 ± 1.2	22.7 ± 1.0	14.3 ± 0.6
Mean oocyst intensity			85.6			19.8
Reduction in oocyst intensity (%)^d^						76.9^***^
Sporozoite/SG^e^	130,000	110,241	141,530	2,994	6,875	1,985
Mice positive after 3,000 sporozoite injection	3/3	3/3	3/3	2/3	3/3	3/3
Day of 0.5-2% parasitemia after sporozoite injection	5.3	5	5.7	6.5	5.7	5

^a^ The prevalence of infection was determined using the formula: The number of mosquitoes containing oocysts/the total number of mosquitoes dissected × 100%.

^b^ The percent reduction in prevalence was calculated using the formula: the mean prevalence_WT_ - the mean prevalence_Δ*pbpp6*_; Fisher’s exact test; *** *P* < 0.001;

^c^ The number of oocysts per mosquito midgut (mean ± SEM);

^d^ The percent reduction in oocyst intensity was calculated using the formula: (mean oocyst intensity_WT_ − mean oocyst intensity_Δ*pbpp6*_)/ mean oocyst intensity_WT_ × 100%; Mann-Whitney *U* test; *** *P* < 0.001;

^e^ The mean number of salivary glands examined was 10.

With such a transmission reducing effect of *pbpp6* deletion, we wondered whether inhibition of the PbPP6 enzyme activity would have a similar effect. We assessed the exflagellation and *in vitro* ookinete conversion of WT *P. berghei* in the presence of okadaic acid. At the final concentration of 1 × IC_50_ (0.86 nM) and 1.5 × IC_50_ (1.29 nM), okadaic acid dramatically inhibited exflagellation and ookinete conversion in a concentration-dependent manner (S5 Fig).

### Integrated omics analysis of Δ*pbpp6* gametocytes

To elucidate the molecular mechanisms underlying the gametogenesis defect in the Δ*pbpp6* line, transcriptomic and proteomic analyses were performed on activated gametocytes (15 min post-activation). High reproducibility among biological replicates was confirmed by hierarchical clustering of RNA-seq read counts (S1A Table) and Pearson correlation coefficients of proteomic replicates (WT: *r* = 0.81–0.85; Δ*pbpp6*: *r* = 0.85; S7A Fig and S2A Table).

Transcriptomic analysis identified 493 significantly altered transcripts (151 up-regulated, 342 down-regulated; adjusted *P*-value < 0.1, |log₂FC| > 1) out of 5,159 detected genes (S6A Fig and S1B–C Table). Down-regulated genes included those involved in gametogenesis and transmission, such as *NEK3* [19–21], *MDV1* [22], and LCCL proteins (*LAP5*, *CCp1*) [23], as well as *AP2-Z*, *IMP4*, and *P25/28*, all exhibiting fold changes greater than 4.8 (S6B Fig and S1C Table). GO enrichment analysis revealed that up-regulated transcripts were associated with protein homeostasis and ribosomal functions, whereas down-regulated transcripts were linked to cellular signaling processes (specifically *cyclic nucleotide metabolic process* and *cyclic-nucleotide phosphodiesterase activity*), motility, and cytoskeletal organization (S6C Fig and S1D Table). Reduced expression of phosphodiesterases (PDEα, γ, δ) (S1C Table) indicated disruption of the cGMP-PKG-Ca^2^⁺ signaling cascade in Δ*pbpp6* gametocytes, potentially explaining the observed developmental deficiency.

Proteomic analysis identified 1,896 *P. berghei* proteins, with 327 differentially expressed (162 up-regulated, 165 down-regulated; |log₂FC| > 0, *P*-adj < 0.1; S7B–C Fig and S2C Table). Up-regulated proteins included gamete egress regulators (MDV1, GEST, MiGS, MAPK2, G377, GEP) [24–26], while down-regulated proteins were implicated in endocytosis, DNA metabolism, phosphorylation, and inner membrane complex formation (KIC transporters, histones, CDPK6, IMC components) [7,27–29] (S7D Fig and S2C Table). Sex-specific analysis revealed 51.1% shared dysregulation, 8.3% (27/327) male-specific and 10.1% (33/327) female-specific dysregulation (S2C Table). Critically, significant alterations were observed for the female-specific transcription factors AP2-FG (0.8-fold downregulation) and PFG/FD2 (1.2-fold upregulation). GO analysis highlighted enrichment of up-regulated proteins in ribosome-associated processes and protein folding, while down-regulated proteins were predominantly membrane-associated (S7E Fig and S2D Table).

Comparative analysis of transcriptomic and proteomic revealed a moderate correlation between transcript and protein levels (Spearman’s *r* = 0.28, *P* = 2.2e-16; S8A Fig), with only 41.4% (1,300 genes) of transcripts (FPKM >20 in either the WT or Δ*pbpp6* across triplicates) corresponding to detectable proteins (S8B Fig). The remaining 58.6% (1,840 genes) included female-enriched, translationally repressed transcripts, such as *pbs28*, *plasmepsin* (*pm*)8/9, *cpw-wpc* family members, palmitoyl-S-acyltransferases proteins *dhh3/10*, *rom3*, and *trap*, representing 45.6% of the known *Plasmodium* TR transcripts [30,31] (S3A–B Table). Stabilization of these TR genes requires the mRNP complex in gametocytes [32]. Notably, 13 out of the 16 major subunits of the mRNP particle were identified in our proteomic analysis (the transcript-protein correlation: *r* = 0.05, *P* = 2.29e-11). With the exception of HoMu protein, all detected mRNP subunits were up-regulated upon *pbpp6* deletion (S2B Table and S8C Fig), providing insight into the mechanisms of translational dysregulation.

### *Pbpp6* disruption impairs the cGMP-PKG-Ca^2+^ signaling pathway during gametogenesis

**Fig 4:**
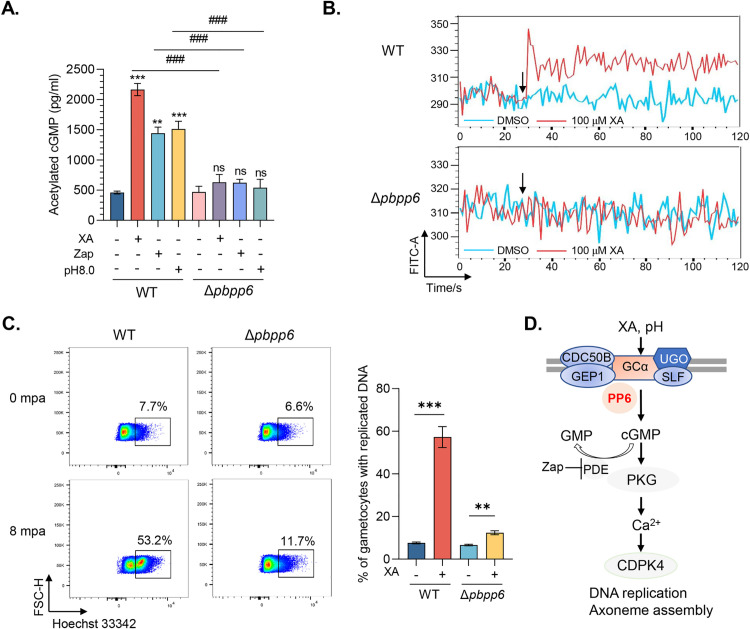
PbPP6 disruption impairs the cGMP-PKG-Ca^2+^ signaling pathway. A. The cGMP levels in WT and Δ*pbpp6* gametocytes after stimulation with DMSO (control), 100 μM xanthurenic acid (XA), 100 μM zaprinast (Zap), or pH 8.0, measured 2 min post-treatment (n = 3). Data represent mean ± SD (three biological replicates). Statistical significance: ns (not significant), ** *P* < 0.01, *** *P* < 0.001 vs. respective controls; ### *P* < 0.001 for WT vs. Δ*pbpp6* (Student’s *t*-test). B. Cytosolic Ca^2^⁺ kinetics in Fluo-8-loaded gametocytes monitored by flow cytometry for 30 s before and 90 s after XA/DMSO treatment. Signals were normalized to baseline Ca^2^⁺ levels in DMSO controls. Black arrows indicate treatment timepoints. Representative data from three biological replicates are shown. C. Genomic DNA replication in XA-activated gametocytes (8 min post-treatment) assessed by Hoechst 33342 fluorescence. The data illustrate the proportion (left) and percentage of gametocytes with replicated DNA (right). Representative data from three biological replicates are shown. Statistical comparisons within the WT and Δ*pbpp6* groups indicated significance, with ** *P* < 0.01 and *** *P* < 0.001 for XA- vs. DMSO-treated groups (Student’s *t*-test). D. A hypothetical location of PbPP6 in the cGMP-PKG-Ca^2+^ signaling cascade during gametogenesis is illustrated.

The cGMP-PKG-Ca^2+^ signaling cascade is vital for gametogenesis [33]. The deletion of the *pbpp6* gene disrupts several components of this cascade, prompting us to investigate the role of PbPP6 in this signaling pathway. We compared intracellular cGMP concentrations in activated gametocytes from WT and Δ*pbpp6* lines. Treatment with XA, the phosphodiesterase (PDE) inhibitor zaprinast, or a pH of 8.0 significantly increased cGMP levels in WT gametocytes but not in the Δ*pbpp6* line, suggesting that PbPP6 is involved in cGMP synthesis (Fig 4A). Additionally, we observed a roughly 1.2-fold increase in the cytosolic Ca^2+^ signal in WT gametocytes approximately 5 seconds after XA stimulation. However, this response was absent in the Δ*pbpp6* line. This indicates that the deletion of *pbpp6* impairs downstream Ca^2+^ mobilization during gametogenesis (Figs 4B and S9A). To further investigate whether the deletion of *pbpp6* affects genome replication during male gametogenesis, we analyzed the DNA content in gametocytes after XA induction using flow cytometry. In XA-treated WT parasites, we noted an increase in fluorescence signal from 7.6% to 57.3% (Student’s *t*-test, *P* < 0.001; Figs 4C and S9B). In contrast, the Δ*pbpp6* line showed only a minor increase, from 6.6% to 12.4% (Student’s *t*-test, *P* < 0.01; Figs 4C and S9B). Collectively, our results demonstrated that parasites with disrupted PbPP6 cannot efficiently generate cGMP, leading to severe impairment in downstream Ca^2+^ signaling during gametogenesis (Fig 4D).

### Phosphoproteomic analysis of PbPP6-regulated signaling during gametogenesis

**Fig 5:**
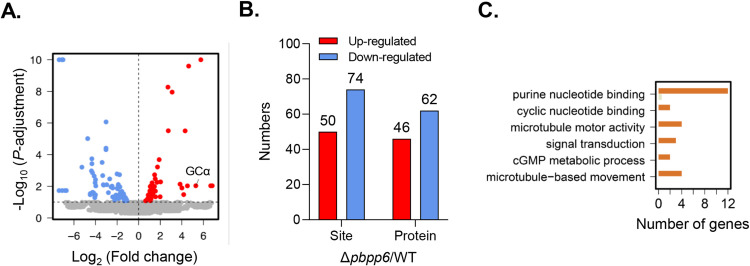
Quantification of phosphosites of Δ*pbpp6* parasites at gametocyte stages. A. The volcano plot illustrates the significantly regulated phosphorylation sites (red, up-regulated phosphorylation sites; blue, down-regulated phosphorylation sites) upon PbPP6 deletion. Refer to S4A Table. B. Quantification of the numbers of regulated phosphosites and proteins in the Δ*pbpp6* strain compared to the WT strain. Refer to S4A Table. **C.** GO analysis of significantly up-regulated phosphorylation proteins in the Δ*pbpp6* strain compared to the WT strain. Related to S4B Table.

To identify the potential perturbation of protein phosphorylation upon *pbpp6* deletion during gametogenesis, we conducted a quantitative phosphoproteome analysis of activated WT and Δ*pbpp6* gametocytes, focusing on samples taken 2 min post-activation. Equal amounts of protein extracts from both WT and Δ*pbpp6* gametocytes were subjected to trypsin digestion and phosphopeptide enrichment, and TMT-labeled peptides were quantified by LC-MS/MS (S4 Table). After normalization of the TMT-labeled proteomic data, 9935 unique peptides were identified, of which 6955 were phosphopeptides. These phosphopeptides contained 9490 phosphosites, of which 6374 were quantifiable, and mapped to 1717 proteins at site level with confidence > 0.75 [34] (S4A Table). By selecting log_2_FC higher or lower than 0 consistently in all biological replicates and average adjusted *P*-value < 0.1, we identified 50 up-regulated phosphosites on 46 proteins and 74 down-regulated phosphosites on 62 proteins, referred to herein as regulated sites (Fig 5A–B and S4A Table). GO analysis of the proteins with significantly up-regulated phosphosites revealed several biological process terms, including *microtubule-based movement*, *cGMP metabolic process*, and *signal transduction*. Additionally, we identified molecular function terms such as *microtubule motor activity*, *cyclic nucleotide binding*, and *purine nucleotide binding* (Fig 5C and S4B Table). Notably, the enriched GO terms for *cyclic nucleotide metabolic process* and *cyclic-nucleotide phosphodiesterase activity* included up-regulated phosphosites in proteins such as guanylyl cyclase alpha (with a ~ 39-fold increase at Ser508) and PDEα (with a ~ 16-fold increase at Ser494), which are involved in the synthesis and hydrolysis of cGMP during gametogenesis (Fig 5 and S4 Table). Overall, these findings indicate that the deletion of *pbpp6* disrupts the phosphorylation status of proteins involved in cGMP synthesis and hydrolysis within the cGMP-PKG-Ca^2+^ signaling pathways during gametogenesis.

## Discussion

We identified a critical role of a novel protein phosphatase, PbPP6, in sexual development of the malaria parasite *P. berghei*. It has been reported that the PbPP6 homologue in *Toxoplasma gondii*, TgPP6C regulates parasite replication and virulence [15]. Although the sequence identity of the PP2Ac domain between these two proteins is high, the low sequence identity of full-length protein between PbPP6 and TgPP6C implies that PbPP6 may function distinctly from TgPP6C, probably by interacting with different substrates. Our previous study in assessing the transmission blocking activity of 25 phosphatase inhibitors showed that two phosphatase inhibitors, BVT-948 and alexidine dihydrochloride, significantly inhibited sexual stage development from gametogenesis to ookinete maturation, indicating that phosphatases can be considered as promising targets for drug development [35]. A PP2A family inhibitor okadaic acid showed potent inhibition of the rPbPP6 enzyme and significant transmission-blocking activity, suggesting that *Plasmodium* PP6 is a promising target for disrupting malaria transmission. It is encouraging that two natural products, fostriecin and LB100, acting as highly selective inhibitors for PP2Ac, have entered phase I human clinical trials for anti-tumor treatment [36,37]. Further studies may alleviate the general toxicity of PPP family inhibitors to all eukaryotic cells and lead to the development of highly selective and specific inhibitors of PPPs targeting the parasite development.

Deletion of *pbpp6* had a profound effect on gametogenesis, a key step within mosquitos that is essential for malaria parasite transmission. After a blood meal by mosquito, gametogenesis is triggered by temperature drop and the presence of xanthurenic acid (XA) in mosquito [38,39]. It has been well-established that cGMP-PKG-Ca^2+^ signaling cascade controls XA-triggered gametogenesis event in *Plasmodium* species [33,40–42]. In the present work we obtained clear evidence that PbPP6 disruption impairs cGMP-PKG-Ca^2+^ signaling pathway in male gametogenesis. Our global transcriptomic analysis also revealed that the down-regulated transcripts of Δ*pbpp6* mutant were enriched in GO terms related to signaling transduction (*cGMP metabolic process* and *cyclic nucleotide metabolic process*), and within these GO terms, we observed components of cGMP-PKG-Ca^2+^ signaling cascade, including PDEα, PDEγ, and PDEδ. Of these, PDEδ is predominantly expressed in gametocyte stages and has a specific cGMP hydrolytic activity [43]. Consistent with the gametogenesis deficiency observed in Δ*pbpp6* mutants, previous reports showed that the activation of male gametocytes is dramatically reduced in PDEδ^–^ mutant [43]. Once the cGMP-PKG-Ca^2+^ signaling cascade is activated, the differentiation of male gametocytes into gametes results in DNA replication, followed by parasitophorous vacuole membrane (PVM) rupture and gametes egress from the host cell [44]. Our proteomic analysis revealed a compensatory upregulation of egress-related effectors in Δ*pbpp6* parasites, including MDV1 and GEST, which are responsible for mediating PVM disruption [22,45]; G377 and MiGS, which are essential for biogenesis of osmiophilic bodies and gamete egress [45–47]; and GEP, which plays a role in executing PKG-dependent egress steps [25,48]. This coordinated proteomic shift suggests a compensatory mechanism due to impaired PKG-Ca^2^⁺ signaling activity. A critical step in the release of male gametes is the activation of a male-specific protein kinase known as MAPK2, which acts subsequent to CDPK4 during cytokinesis, chromatin condensation, and axoneme motility in male gametogenesis [20,49]. MAPK2 activation is known to be modulated by Nek3 kinase [19–21], whose transcript levels were significantly reduced in Δ*pbpp6* parasites according to our RNA-seq analysis. While the direct transcriptional regulation of PKG-Ca^2^⁺ signaling components by PbPP6 remains unverified, both Nek3 and MAPK2 have been established as downstream effectors of PKG-mediated Ca^2^⁺ mobilization during gametogenesis and ookinete gliding [40,50]. Therefore, the dyregulation of these proteins in Δ*pbpp6* parasites likely reflects secondary effects of compromised cGMP-PKG-Ca^2^⁺ signaling.

Interestingly, we found that the phosphorylation levels of both the Ser508 site in GCα and the Ser494 site in PDEα were significantly increased in Δ*pbpp6* gametocytes just 2 minutes after activation. The levels of cGMP, which are crucial for initiating gametogenesis, are tightly controlled by GCs and PDEs. A previous study found that PDEα and PDEδ do not directly raise cGMP levels in response to XA or pH changes. Instead, they play a role in preventing the premature activation of gametocytes in the vertebrate host at 37°C [43]. In contrast, GCα is the primary enzyme that synthesizes cGMP in response to XA or elevated extracellular pH [43]. Previous research has shown that phosphorylation plays a key role in regulating the activity of soluble GCs [51,52]. Meanwhile, a recent research on *P. falciparum* shows that PP1 regulates the activity of GCα during merozoite egress [53], indicating that protein phosphatases play a role in the regulation of cGMP-PKG-Ca^2+^ signaling pathways in malaria parasites. Therefore, we hypothesized that the knockout of PbPP6 leads to hyperphosphorylation and functional deregulation of GCα, ultimately resulting in decreased cGMP production. The observed decrease in calcium mobilization in the Δ*pbpp6* line is likely a secondary effect rather than a direct consequence of PbPP6 disruption. We propose that the use of a cGMP analog could help bypass this blockage and allow us to further test our hypothesis in future studies. As GCα functions as a membrane-anchoring protein, PbPP6 may associate with the plasma membrane through a transient or indirect interaction with GCα during gametogenesis. However, biochemical validation through co-immunoprecipitation and membrane fractionation remains essential to identify PbPP6 interactors. Collectively, these findings highlight the role of the serine/threonine phosphatase PbPP6 in regulating cGMP-PKG-Ca^2+^ signaling pathways during gametogenesis.

The ApiAp2 family contains the APETALA-2 (AP2) DNA binding domain that recognizes multiple and distinct palindromic DNA sequences, which accounts for tighter regulation of many stage-specific gene expressions [54]. It has been reported that two female-specific transcription factors (TFs), AP2-FG and PFG (FD2) form a complex and regulate a broad repertoire of target genes, which are involved in fertilization, meiosis, and ookinete development, such as AP2-Z that regulates zygote development [54–57]. Disruption of either of these two TFs could impair female gametocyte development, and produce abnormal morphology and reduced number of ookinetes [55,56]. In our proteomic analysis, we observed dysregulated expression of both two TFs. As genes under the control of these two TFs partially overlapped, the down-regulated expression of AP2-FG may be responsible for the female gametogenesis deficiency observed in the *pbpp6* deletion parasites, while the upregulated expression of PFG maybe a compensatory mechanism for parasite to complete its sexual stage development. However, the relatively modest (~20% variation) expression shifts may also reflect biological variation or secondary effects of impaired gametogenesis. To determine whether TF dysregulation directly drives the gametogenesis deficiency in PP6-deficient parasites, further studies, including complementation assays and mechanistic investigations, will be necessary in future analysis.

In addition to the functions of PbPP6 during gametogenesis, *pbpp6* deletion also affected post-fertilization ookinete development. Consistently, several genes including *kinesin-13, -20* and *pbs25/28* that are involved in zygote/ookinete development, midgut epithelium traversal and oocyst development [58,59], were also down-regulated upon *pbpp6* deletion. In gametocytes, we observed highly abundant transcripts without proteome evidence, which is consistent with the presence of a large set of TR transcripts in female gametocytes of both *P. falciparum* and *P. berghei* [30,31]. Some of the down-regulated putative TR transcripts (e.g., *pbs28*, *dhhc* and *cpw-wpc* family members) have been shown to be essential for zygote/ookinete development [59–61]. In addition, TR transcripts include genes (e.g., *pm8/9*, *rom3* and *trap*), whose disruption results in a phenotypic effect on oocyst and/or sporozoites development [62–64]. CDPK1 was shown to be important for translation derepression during ookinete development [65]. Although not significant, we found down-regulated expression of CDPK1 in activated gametocyte of *pbpp6* deletion parasites, suggesting the translational regulation of sexual stage-specific mRNA expression may also be affected. Translational repression is a major and conserved mechanism in post-fertilization development, and the storage of these TR transcripts in female gametocytes is known to require mRNP complex, whose two core components DOZI and CITH play an essential roles in ookinete motility [32]. We have observed disturbance of the mRNP complex upon *pbpp6* deletion, which includes upregulation of 12 out of 13 detected mRNP subunits and down-regulation of HoMu [31]. In neural stem cells, HoMu inhibits translation initiation by competing with eIF4G for poly(A) binding protein (PABP)-binding [66]. Therefore, there is a possibility that the overall disturbance of the mRNP complex and reduced expression of HoMu could affect mRNP function in female gametocytes, leading to post-fertilization development deficiency in the mosquito. Alternatively, the apparent upregulation of mRNP proteins in the Δ*pbpp6* strain may simply be a downstream effect of incomplete gametogenesis. In WT strain, these proteins are likely rapidly degraded upon activation, which releases translational repression. However, in the Δ*pbpp6* strain, this degradation might not occur due to the incomplete gametogenesis, leading to an apparent increase in protein levels when comparing the knockout (KO) and WT strains, even if the actual levels haven’t changed. Additionally, deletion of *pbpp6* resulted in reduced mosquito infection rates and oocyst densities. The reduced oocyst numbers are consistent with the observed defect in ookinete conversion and likely represent a follow-up phenotype to this earlier impairment.

## Conclusion

Our results identified PbPP6 as a crucial factor in controlling several essential biological steps in the life cycle of malaria parasites. Although the precise function of PbPP6 in sexual stage development remains to be solved and the molecular targets of PbPP6 need to be verified, it is tempting to speculate that PbPP6 plays a critical function through regulating the function of GCα in male gametogenesis and possibly midgut invasion of ookinetes. Meanwhile, by altering the translation profile of mRNP, PbPP6 may play a role in regulating female gametogenesis and the following post-fertilization development in the mosquito. It is now widely accepted that drugs targeting both asexual and sexual stages are highly desirable for malaria control [67]. Although deletion of PbPP6 has only a mild effect on asexual blood-stage growth, it severely impairs parasite transmission in the mosquito. Therefore, targeting PbPP6 activity holds promise primarily as a strategy to block parasite transmission. Further elucidation of PbPP6’s involvement in signaling pathways could lead to novel transmission-blocking strategies, and the determination of substrates of PbPP6 is therefore a logical next step.

## Materials and methods

### Ethics statement

All animal experiments were approved by the Institutional Animal Care and Use Committee (IACUC) of China Medical University (Approval No. CMU2022189) and performed in compliance with institutional ethical guidelines for animal research.

### Parasite and mosquito maintenance

The *Plasmodium berghei* ANKA parasites were propagated in female Kunming or BALB/c mice (BEIJING HFK BIOSCIENCE Co., Ltd., China), aged 6–8 weeks [68]. Colonies of *Anopheles stephensi* were maintained under controlled conditions: 25°C, 50–80% relative humidity, and a 12-hour photoperiod 12-hour light/dark cycle. Adult female mosquitoes were provided with 10% (w/v) glucose solution [68].

### Sequence analysis

The PP6 sequences in *P. falciparum* (PfPP6, PF3D7_0314400) and *P. berghei* (PbPP6, PbANKA_041210) were retrieved from PlasmoDB (plasmodb.org); PP6 sequence in *Toxoplasma gondii* (TgPP6, TGARI_301010) was retrieved from ToxoDB (toxodb.org); PP6 sequence in *Cryptosporidium parvum* (CpPP6, GY17_00001243-t36_1) was retrieved from CryptoDB (cryptodb.org); and PP6 homologs in *Homo sapiens* PP6C (HsPP6C, NP_002712.1), *Drosophila melanogaster* PPV (DmPPV, NP_511061.1), *Saccharomyces cerevisiae* SIT4 (ScSIT4, NP_010236.1) and *Schizosaccharomyces pombe* Ppe1 (SpPpe1, NP_588420.1) were retrieved from GenBank. The conserved domains and motifs of PbPP6 were analyzed using the SMART software (http://smart.embl-heidelberg.de/) and the Motif Scan software (https://myhits.isb-sib.ch/cgi-bin/motif_scan) [69]. Sequence alignment was performed with MUSCLE software (https://www.ebi.ac.uk/Tools/msa/muscle/). The BioEdit Sequence Alignment Editor was used for visualization of the alignment. For the phylogenetic analysis of PP6 sequences, the Neighbor-Joining method in MEGA 7.0 was employed and modified using Eolview v3 [70,71].

### Recombinant protein expression and antisera production

The complete open reading frame (ORF) of PbPP6, optimized for expression in the *Pichia* expression system, was expressed in the GS115 his4 strain (Genecreate, Wuhan, China). The recombinant PbPP6 protein (rPbPP6) was purified using HisPur Ni-NTA (ThermoFisher, MA, USA). SDS-PAGE was performed on the protein samples to assess their purity, and these samples were subsequently used for immunization in mice according to a standard immunization protocol [68]. A control recombinant Trx-A protein with 6 × His tag that was present on the pET-32a (+) plasmid was expressed and purified as previously described [10]. Rabbit anti-PbCDPK1 sera were custom-made against recombinant PbCDPK1 protein (amino acid position: 260 – 523) by Genecreat (Wuhan, China).

### PbPP6 phosphatase assay

The recombinant PbPP6 (rPbPP6) phosphatase activity was measured using the ProFluor Ser/Thr PPase Assay kit (Promega, WI, USA). rPbPP6 and a His-tagged control protein were diluted to a concentration of 30 nM and mixed with the Peptide Solution (containing 5 × Reaction Buffer B [200 mM Tris-HCl, pH 7.5, 0.5 mg/ml BSA] and S/T PPase R110 Substrate [10 mM]) for 15 seconds, followed by incubation at room temperature (RT) for 10 minutes. Protease Solution was subsequently added, and the mixture was incubated at RT for an additional 90 minutes. Following the addition of Stabilizer Solution, fluorescence was measured using a Biomek 2000 workstation (Beckman Coulter, CA, USA) with an excitation wavelength of 485 nm and an emission wavelength of 530 nm. Inhibition assays using okadaic acid were conducted as described [10].

### Western blot and immunofluorescence assays

Parasites at various developmental stages (ring, trophozoite, schizont, gametocyte, and ookinete) were purified as described [72]. Briefly, mice pretreated with phenylhydrazine (6 mg/ml) were intravenously injected with 1 × 10^8^ parasites. At 3 days post-infection (d.p.i.), sulfadiazine (20 mg/ml, Sigma, MO, USA) was administered in drinking water for 48 hr to eliminate asexual-stage parasites. Gametocytes were then isolated through cardiac puncture and separated from uninfected erythrocytes using a 48% NycoDenz gradient. Schizonts and ookinetes were obtained by culturing the infected blood at 37°C overnight and at 20°C for 24 hr, respectively, followed by purification on 55% or 62% NycoDenz gradients. For the ring and trophozoite stages, purified schizonts were injected intravenously into naïve mice, and the parasites were collected at 4 hr (for rings) or 12 hr (for trophozoites) post-invasion after two replication cycles.

For protein extraction, purified parasites were lysed using 0.15% saponin (Sigma) in PBS. After centrifugation at 13,000 × g for 5 min, the samples were washed with PBS. Proteins were extracted using M-PER Mammalian Protein Extraction Reagent (Thermo Fisher) and quantified with the Pierce BCA Protein Assay Kit (Thermo Fisher), followed by resolution via 10% SDS-PAGE. Western blotting was performed as described [10] using mouse anti-PbPP6 sera, anti-GFP mAb (Abcam, Cambridge, UK), or anti-Hsp70 mAb (Abcam), followed by HRP-conjugated goat anti-mouse IgG (H + L) antibodies (Thermo Fisher). Signal detection was accomplished using SuperSignal West Pico PLUS (Thermo Fisher) on a Tanon 4200 imaging system (Tanon, Shanghai, China).

For the solubility analysis, ookinetes from PbPP6-GFP parasites were lysed in 5 mM Tris-HCl (pH 8.0) and then frozen at −80°C [73]. After thawing, soluble proteins were collected through centrifugation at 16,000 × g for 10 min. The resulting pellet was resuspended in 0.1 M Na_2_CO_3_ and incubated on ice for 30 min to extract peripheral membrane proteins. The proteins were then resolved by SDS-PAGE and probed with anti-GFP mAb, anti-GAPDH (Abcam), or anti-CDPK1 serum.

For immunofluorescence assays (IFA), both WT and PbPP6-GFP parasites were subjected to fixation utilizing a solution composed of 4% paraformaldehyde (PFA) and 0.0075% glutaraldehyde in PBS. Following fixation, the samples were permeabilized with 0.1% Triton X-100 for 5 min on ice, and subsequently neutralized with NaBH_4_ at a concentration of 0.1 mg/ml. After blocking with 5% non-fat milk, the samples were incubated with a series of primary antibodies: mouse anti-PbPP6 (1:500), rabbit anti-GAPDH, rabbit anti-MSP1 (1:500), rabbit anti-CDPK1 (1:500), rabbit anti-Pbg377 (female marker, 1:500), rabbit anti-α-tubulin II (male marker, 1:500), mouse anti-GAP40 (glideosome marker, 1:500), rabbit anti-GFP mAb (Abcam), and mouse anti-Pbs21 (ookinete marker, 1:500). Secondary antibodies conjugated to Alexa Fluor 488 and 594 (1:500, Thermo Fisher) were subsequently employed. The samples were then mounted with ProLong Diamond Antifade Mountant containing DAPI for nuclear counterstaining. Imaging was conducted using a Nikon C2 confocal microscope, and data analysis was performed using ImageJ

### Generation of transgenic parasites

The *pbpp6* gene in *P. berghei* parasites was disrupted in two independent experiments using either a double- or single-crossover homologous recombination strategy [74,75]. For the double-crossover approach, a 1460 bp fragment of the 5′ untranslated homologue recombinant region (5R) (-1502 – -71 bp) and an 1157 bp fragment from the 3R (33 bp – 1162 bp) of the *pbpp6* gene were amplified using the parasite genomic DNA as a template (primers are listed in S5 Table), and cloned into the HindIII/PstI and XhoI/NotI sites of the pL0034 vector, which contains the h*dhfr* selection cassette, resulting in the creation of the pL0034-PbPP6KO plasmid. For the single-crossover approach, amined at disrupting and C-terminally tagging PbPP6 with a 2 × FKBP followed by GFP (referred to as dPbPP6), a 408 bp fragment (25–432 bp) from the *pbpp6* open reading frame (ORF) and an 898 bp fragment (26–924 bp) were amplified and cloned into the NotI/AvrII sites of pSLI-2 × FKBP-GFP vector [75] to generate the pSLI-dPbPP6–2 × FKBP-GFP and pSLI-PbPP6tag-2 × FKBP-GFP plasmids, respectively.

Transfection, selection and cloning of transgenic parasites were performed using genetic modification technology developed for *P. berghei* [74,76]. Resistant parasites were selected by 70 mg/L pyrimethamine (Sigma) supplied in the drinking water for 7 days. For *pbpp6* gene disruption and C-terminal tagging with GFP, resistant parasites selected by pyrimethamine were adjusted to 1% parasitemia and further selected by intraperitoneal injection of G418 (Sigma) at 40 mg/kg. Successful integration of the plasmids was confirmed by genotyping PCR using primers in S5 Table.

### Phenotype analysis

The phenotypic characterization of transgenic parasite lines (Δ*pbpp6* K1, Δ*pbpp6* K2, PbPP6-GFP C1) and WT-infected RBCs (iRBCs) was injected into 6–8-week-old BALB/c mice. Daily parasitemia was monitored by Giemsa-stained tail blood smears, with concurrent documentation of mouse survival rates. At 3 dpi, gametocytemia was quantified by enumerating mature gametocytes per 1,000 RBCs, and the male-to-female gametocyte ratio was determined using Giemsa-stained smears.

To evaluate gamete formation rates, blood samples were activated with a gametocyte activation buffer (GAB, RPMI 1640, penicillin, streptomycin, 20% fetal calf serum, heparin, and 100 μM XA, pH 7.4) for 30 min at 25°C, followed by sex-specific staining (anti-α-tubulin II/Ter-119 for male gametes and anti-Pbg377/Ter-119 for female gametes). Gametes were considered to have egressed/not-egressed if they were negative/positive for Ter119 staining. Exflagellation centers and gamete interactions were quantified as described [10].

The ookinete conversion was assessed in *vitro* using Pbs21 surface marker staining [72,77]. The mosquito transmission experiments were performed by feeding 30–60 *An. stephensi* per replicate on infected mice, with oocyst counts at 10 days post-feeding [78]. Gamete fertility was evaluated through crosses with Δ*p47* (female-defective) or Δ*p48/45* (male-defective) parasite lines [10]. For the sporogonic development analysis, 3,000 salivary gland sporozoites (from either WT or Δ*pbpp6* strains) were injected intravenously into BALB/c mice at a dosage of 100 μL, with parasitemia monitored via Giemsa-stained blood smears from day 4 post-injection. The exflagellation and ookinete conversion inhibition assays were performed as described [10]. Each assay included three biological replicates to ensure valid and reliable results.

### Flow cytometry assay

Cytosolic Ca^2^⁺ mobilization was assessed as described [25,79]. Purified WT and Δ*pbpp6* gametocytes were incubated with 5 μM Fluo-8 (ATT Bioquest, CA, USA) in calcium-free buffer (CFB: 137 mM NaCl, 4 mM KCl, 20 mM glucose, 20 mM HEPES, 4 mM NaHCO₃ [pH 7.2-7.3], 0.1% BSA) for 20 min at 37°C. Following three washes with CFB, baseline fluorescence was recorded for 30 sec on a FACS Celesta flow cytometer. Subsequently, the samples were stimulated with 100 μM xanthurenic acid (XA, Sigma) and fluorescence measurements were taken for an additional 90 sec. The data were analyzed using FlowJo 10 software (BD Pharmingen, NJ, USA) to ensure precise quantification of cytosolic Ca^2^⁺ dynamics.

For DNA content analysis, gametocytes were either subjected to immediate fixation with 4% PFA or were activated in a GAB buffer for 8 min at 25°C prior to fixation. The fixed samples were stained with Hoechst 33342 for 30 min at 37°C and were subsequently analyzed using a FACS Celesta flow cytometer with FlowJo v10.8.1 software (BD Pharmingen).

### Measurement of cellular cGMP levels

The intracellular concentrations of cGMP in WT and Δ*pbpp6* gametocytes were measured following stimulation with XA as described [79]. Briefly, 2 × 10^7^ WT and Δ*pbpp6* gametocytes were purified and incubated for 2 min in gametocyte maintenance buffer (GMB: 137 mM NaCl, 4 mM KCl, 1 mM CaCl_2_, 20 mM glucose, 20 mM HEPES, 4 mM NaHCO_3_, 0.1% BSA, pH 7.24–7.29) supplemented with 100 μM XA, 100 μM Zap, pH 8.0, or DMSO as a vehicle control. Subsequent lysis was performed using ice-cold 0.2 M HCl for 10 min, followed by mechanical disruption through sequential passages in a 27-gauge needle. The cGMP levels were determined using a cGMP ELISA kit (Cayman Chemical, MI, USA), following the acetylated protocol.

### Transcriptome analysis of gametocytes

Total RNA was extracted from activated gametocytes (25°C for 15 min) of both Δ*pbpp6* and WT parasites using the Qiagen RNeasy kit (Qiagen, Dusseldorf, Germany). The quantity, purity and integrity of the RNA were assessed through 1.5% agarose gel electrophoresis and analyzed with a Nanodrop OneC spectrophotometer (ThermoFisher). Stranded mRNA-seq libraries were prepared from 2 μg of total RNA per sample using the KC-Digital Stranded mRNA Library Prep Kit (Wuhan Seqhealth Co., Hubei, China) [80]. RNA-seq was conducted using three biological replicates of both the Δ*pbpp6* and WT parasites. The library products were sequenced on a Hiseq X 10 sequencer (Illumina, San Diego, USA).

Raw sequencing data were filtered using Trimmomatic (v0.36) and processed with in-house scripts to eliminate duplication bias introduced during library preparation and sequencing. The RNA-seq reads from each sample were aligned to the PlasmoDB-67 reference genome of the *P. berghei* ANKA using the STAR RNA-seq alignment tool [81]. The HTSeq tool (v.0.6.1) was employed to count the transcripts for each gene [82]. Normalization was performed using Cuffdiff (v2.1) [83], and differential expression analysis was conducted with DESeq2 (v.1.28.1) in R (v.4.04) [84]. Genes with an false discovery rate (FDR) below 0.1 and a minimal log_2_Fold change (log_2_FC) of 1 were considered significantly differentially expressed. Clustering and PCA were carried out in R to estimate sampling distribution for each experiment. GO analysis for differentially expressed genes was performed in PlasmoDB [85], with an adjusted *P*-value threshold of less than 0.1 to determine statistically significant enrichment. The raw RNA-seq data has been deposited in the GEO database (accession number: GSE271105).

### Quantitative proteomic and phosphoproteomic analyses

Proteins from purified activated gametocytes (15 minutes post-activation) were extracted by sonicating in 8 M urea, which was followed by reduction with 5 mM DTT at 56 °C and alkylation with 11 mM iodoacetamide at room temperature. The sample then underwent overnight digestion with trypsin, followed by desalting and drying. The reconstituted samples in 0.5 M TEAB were incubated with TMT reagent for 2 hours, then desalting and drying were repeated. The samples were separated into 60 fractions using an Agilent 300 Extend C18 column with an acetonitrile gradient. Finally, the fractions were combined into 18 and dried using a SpeedVac concentrator.

For phosphoproteomic analysis, we collected activated gametocyte samples (2 min post-activation). The peptide mixtures were incubated at 37°C for 1 h with Ti-IMAC microsphere (ReSyn Biosciences, Beijing, China) to enrich for phosphopeptides. After centrifuging at 12,000 *g* for 5 min, we washed the microspheres sequentially with 50% acetonitrile/6% TFA and 30% acetonitrile/0.1% TFA to remove non-specific binding. The enriched phosphopeptides were eluated with an elution buffer containing 10% NH_4_OH and then lyophilized.

For LC-MS/MS analysis, peptide fractions were dissolved in solvent A (0.1% formic acid in 2% acetonitrile) and directly loaded onto a reversed-phase analytical column (15 cm in length and 75 μm in diameter). LC was performed using the EASY-nLC 1000 Ultra-Performance Liquid Chromatography (UPLC) system (ThermoFisher). The peptides were subjected to nanoelectrospray ionization, followed by MS/MS in a Q Exactive Plus mass spectrometer (ThermoFisher), which was coupled online with the UPLC. The resulting MS/MS data were processed using Maxquant search engine (v.1.5.2.8) and searched against the PlasmoDB-67 reference database for *P. berghei*, using Trypsin/P cleavage with up to 2 missed cleavages. The mass tolerances were set to 20 ppm for initial and 5 ppm for main searches, with a 0.02 Da tolerance for fragment ions. The FDR was adjusted to less than 1%, and peptide scores were > 40. The MS/MS raw data and peptide details (sequence, unique peptides, spectral counts, sequence coverage, and Mascot scores) were deposited in the ProteomeXchange via the PRIDE database (proteomic accession number: PXD053412; phosphoproteomic asscession number: PXD063164).

### Statistical analyses

For the phenotype analysis of the Δ*pbpp6* parasite line, we conducted statistical analyses using GraphPad Prism. We performed a two-tailed, unpaired Student’s *t*-test to compare various metrics, including parasitemia, exflagellation, gametocyte activation, male-female gamete interactions, and ookinete conversion. To assess survival differences in infected mice, we applied Kaplan–Meier’s survival analysis. Oocyst intensity was analyzed using the Mann–Whitney *U* test, while infection prevalence was evaluated with Fisher’s exact test.

## Supporting information

S1 Fig Sequence analysis of PbPP6 protein. A. Schematic representation highlighting the phosphatase domain (PP2Ac) within the PbPP6 protein. **B.** Multiple sequence alignment of conserved PP2Ac domains across PP6 orthologs, performed using CLUSTALW with manual refinement. Included species: *Plasmodium berghei* PP6 (PbPP6 [Pb], PbANKA_0412100), *Plasmodium falciparum* PP6 (PfPP6 [Pf], PF3D7_0314400), *Toxoplasma gondii* PP6 (TgPP6 [Tg], TGARI_301010), *Cryptosporidium parvum* PP6 (CpPP6 [Cp], GY17_00001243), *Homo sapiens* PP6C (HsPP6C [Hs], NP_002712.1), *Caenorhabditis elegans* PPh6 (CePPh6 [Ce], NP_497714.2), *Drosophila melanoqaster* PPV (DmPPV [Dm], NP_511061.1), *Saccharomyces cerevisiae* SIT4 (ScSIT4 [Sc], NP_010236.1), *Schizosaccharomyces pombe* Ppe1 (SpPpe1 [Sp], NP_588420.1). The conserved catalytic motifs and active-site residues are marked with boxed. **C.** Phylogenetic reconstruction of PP6 orthologs was performed using the neighbor-joining method with 1,000 bootstrap replicates.
(TIF)

S2 Fig Purified rings, trophozoites, schizonts, gametocytes, and ookinetes. *P. berghei* schizonts were cultured and enriched using a 55% Nycodenz cushion (v/v). The purified schizonts were injected intravenously into naïve mice. After 48 h (equivalent to two cell cycles), the infected mice were bled by cardiac puncture at 4 h and 12 h (the third cycle) to collect the ring and trophozoite stages, respectively. Gametocytes were harvested from sulfadiazine-treated mice and enriched on a 48% Nycodenz cushion (v/v) at 37°C. Ookinetes were obtained by culturing gametocyte-infected blood and enriched on a 62% Nycodenz cushion (v/v). The resulting smears were stained with Giemsa. Scale bar = 5 µm.
(TIF)

S3 Fig Phenotype analysis of PbPP6-GFP parasites. **A.** Schematic of C-terminal endogenous *pbpp6* tagging with 2 × FKBP-GFP. Primer positions (p1, p2, p3) are indicated by arrows. **B.** Diagnostic PCR of the PbPP6-GFP C1 clone. Lanes 1–2: wild-type (WT); lanes 3–4: C1 clone. PCR products: p1 + p2 (1092 bp, lanes 1/3); p1 + p3 (1776 bp, lanes 2/4). **C.** Western blot of mixed blood-stage parasites using anti-GFP mAb. PbPP6-GFP is marked (arrow); β-actin served as the loading control. (**D–E**) No significant differences were observed between PbPP6-GFP and WT parasites in (**D**) daily parasitemia or (**E**) mouse survival (n = 9 per group). (**F–G**) PbPP6-GFP parasites exhibited (**F**) comparable ookinete conversion rates in *vitro* and (**G**) similar oocyst numbers per midgut in *Anopheles stephensi* mosquitoes (Mann-Whitney *U* test).
(TIF)

S4 Fig *Pbpp6* gene deletion/disruption and analysis. **A.** Targeted deletion of *pbpp6*. **Upper panel:** Double-crossover replacement strategy. **Lower left panel:** Diagnostic PCR results of *pbpp6* deletion. Lane 1/4: cF1 + cR2 (WT, 4031 bp; Δ*pbpp6* parasite, 5787 bp); Lane 2/5: cF2 + cR2 (1919 bp); Lane 3/6: cF1 + cR1 (2293 bp). **Lower right panel:** Immunoblot of parasite lysates probed with anti-PbPP6 sera (arrow indicates PbPP6); Hsp70 served as loading control. **B.** Single-crossover homologous recombination strategy for *pbpp6* disruption. **Upper panel:** Targeting strategy. **Lower left panel:** Diagnostic PCR results: p1 + p3 (1284 bp, red arrow); p4 + p2 (724 bp, black arrow). **Lower right panel:** Western blot detecting truncated PbPP6 (dPbPP6, ~ 71.5kDa) in Δ*pbpp6* parasites using anti-GFP antibody; Hsp70 confirmed equal loading. **C.** IFA analysis of WT and Δ*pbpp6* parasite of schizont and ookinete stages using anti-PbPP6 sera. **D.** Asexual stage growth curves. Growth of asexual blood stages of the WT and two Δ*pbpp6* clones was monitored daily for 15 days (mean ± SEM; *, *P* < 0.05; **, *P* < 0.01; ***, *P* < 0.001). **E.** Survival analysis of infected mice (n = 9/group) by Kaplan-Meier method (WT vs Δ*pbpp6* K1: χ² = 7.569, *P* = 0.006; WT vs Δ*pbpp6* K2: χ² = 8.676, *P* = 0.003). Data represent three independent experiments.
(TIF)

S5 Fig Effect of okadaic acid (OKA) on exflagellation and ookinete formation. **A.** Effect of OKA on gametocyte exflagellation. **B.** Effect of OKA on ookinete conversion. 1 × IC_50_ = 0.86 nM; 1.5 × IC_50_ = 1.29 nM. *, *P *< 0.05; **, *P *< 0.01; ***, *P *< 0.001.
(TIF)

S6 Fig Global transcriptional analysis for activated gametocytes of Δ*pbpp6* parasites by RNA-seq. **A.** Volcano plot displaying the estimated log_2_ fold changes for Δ*pbpp6* versus WT versus the –log_10_ adjusted *P*-values. Each point represents a *P. berghei* gene from our analysis. The significantly differentially transcribed genes (i.e., genes with a *P*-adj < 0.1 and greater than 2-folds difference) are highlighted in orange (up-regulated genes) and green (down-regulated) colored points, respectively. **B.** Heatmap illustrating the significantly down-regulated male gametogenesis-related genes identified in the current study. Related to S1C Table. **C.** Gene Ontology (GO) analysis was performed on both the up-regulated (upper panel) and down-regulated (lower panel) genes following the deletion of PbPP6. Bar graphs present a generic description of the gene sets associated with these functions. Refer to S1D Table. The expected number of genes in a specific GO term was calculated as the fraction of genes categorized as this GO term in *P. berghei* multiple the number of genes up- or down- regulated.
(TIF)

S7 Fig Distribution of predicted proteins from the *P. berghei* proteomes of activated gametocytes. **A.** Hierarchical clustering of Pearson correlation coefficients shows the relationship between all quantified genes for each pair of samples, representing three biological replicates of WT and Δ*pbpp6* parasite proteomes. **B.** Heatmap displaying of all proteins that exhibit significant changes in *Δpbpp6* gametocytes compared to WT gametocytes. Refer to S2 Table. **C.** The volcano plot demonstrates significantly regulated proteins, with orange indicating upregulated proteins and green indicating downregulated proteins following PbPP6 deletion. Refer to S2C Table. **D.** Heatmap illustrating the significantly up- and down-regulated proteins involved in egress, endocytosis, DNA metabolic process, phosphorylation and inner membrane complex location identified in the current study. Related to S2C Table. **E.** GO analysis of significant differentially-regulated proteins in Δ*pbpp6* strain when comparing WT strain. The up-regulated and down-regulated GO terms are displayed by orange and green color, respectively. Refer to S2D Table.
(TIF)

S8 Fig Comparison of the transcriptome and proteome identified in this study. **A.** Scatter plot showing transcript abundance (log_2_ fold change) versus protein abundance (log_2_ fold change) for genes identified in the transcriptome and proteome. The spearman’s correlation value is displayed in the corner of the plot. **B.** Venn diagrams showing the overlap of genes identified in both the transcriptome and proteome. **C.** Heatmap of gene expression, featuring the identified components of the CITH/DOZI complex on the vertical axis and the strains (WT and ∆*pbpp6*) on the horizontal axis.
(TIF)

S9 Fig Flow cytometry gating strategies for calcium mobilization and genomic DNA replication analysis. **A.** Gating approach for intracellular Ca^2^⁺ flux quantification in activated gametocytes pre-loaded with Fluo-8 AM. The black arrow denotes XA or DMSO addition time point. Data were acquired continuously from 30 s pre-stimulation to 90 s post-stimulation (corresponding to Fig 4B). **B.** Gating methodology for genomic DNA replication analysis in Hoechst 33342-stained gametocytes (corresponding to Fig 4C).
(TIF)

S1 Table Transcriptome analysis of Δ*pbpp6* and WT *P. berghei* gametocytes. **A.** Hierarchical clustering of Pearson correlation coefficients between each pair of samples from three biological replicates. **B.** Raw sequencing counts per gene in ∆*pbpp6* transcriptome analysis. **C.** Transcriptome analysis of significantly 2-fold regulated genes in ∆*pbpp6* parasites. **D.** GO analysis of significantly altered transcripts in ∆*pbpp6* parasites compared to WT. Related to S6C Fig.
(XLSX)

S2 Table Quantitative proteomic analysis of Δ*pbpp6* and WT *P. berghei* gametocytes. **A.** Pearson’s correlation of quantification from all quantified proteins between each pair of samples. **B.** Total list of identified proteins in LC-MS/MS analysis. **C.** List of significant differentially expressed proteins in ∆*pbpp6* parasites as measured by LC-MS/MS analysis. Related to S7 Fig. **D.** GO analysis of significantly dysregulated proteins in ∆*pbpp6* parasites compared to WT. Related to S7E Fig.
(XLSX)

S3 Table The putative translationally repressed (TR) analysis of Δ*pbpp6* and WT *P. berghei* gametocytes. **A.** The putative TR genes identified in our study compared with sex-specific transcriptional regulation in *P. falciparum* [31]. **B.** The overlapped putative TR genes of female gametocytes identified in this study and previous reports in *P. falciparum* [31] and *P. berghei* [30].
(XLSX)

S4 Table Phosphoproteomic analysis of Δ*pbpp6* and WT *P. berghei* gametocytes. **A.** Quantification of phosphosites of Δ*pbpp6* parasites in activated gametocytes (2 min post-activation). **B.** GO analysis of significantly dysregulated phosphoproteins in Δ*pbpp6* strain when comparing WT strain.
(XLSX)

S5 Table Oligonucleotides used for plasmid construction and genotype checking. (XLSX)
