# Supplementary figures and images for: Loss of serine/threonine protein phosphatase 6 severely impairs sexual stage development in malaria parasite Plasmodium berghei
Source: PLoS Pathog. 2025 Jul 7;21(7):e1013318. doi: 10.1371/journal.ppat.1013318 (PMC12233266; doi:10.1371/journal.ppat.1013318)

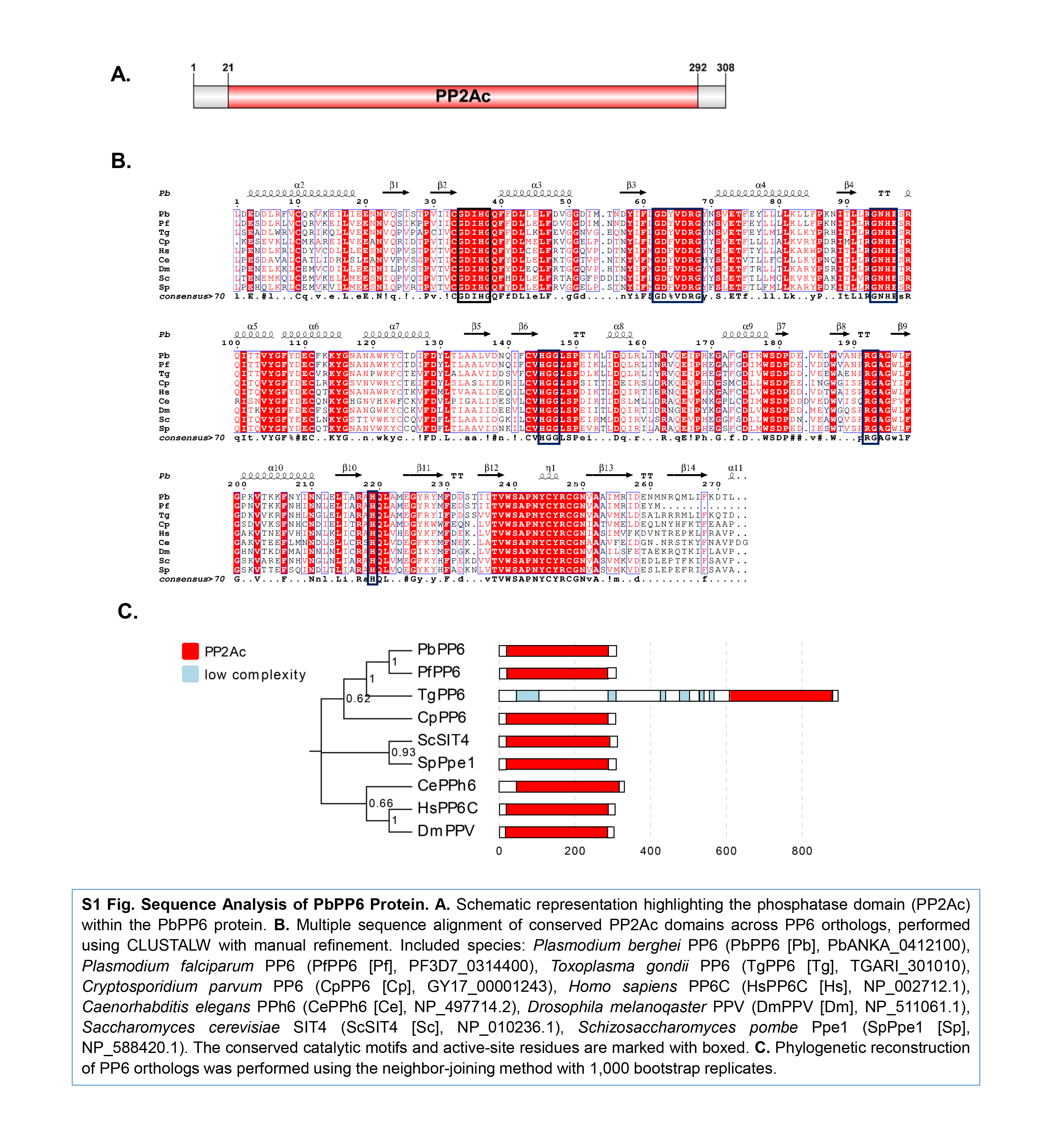

Supplement: S1 Fig — A. Schematic representation highlighting the phosphatase domain (PP2Ac) within the PbPP6 protein. B. Multiple sequence alignment of conserved PP2Ac domains across PP6 orthologs, performed using CLUSTALW with manual refinement. Included species: Plasmodium berghei PP6 (PbPP6 [Pb], PbANKA_0412100), Plasmodium falciparum PP6 (PfPP6 [Pf], PF3D7_0314400), Toxoplasma gondii PP6 (TgPP6 [Tg], TGARI_301010), Cryptosporidium parvum PP6 (CpPP6 [Cp], GY17_00001243), Homo sapiens PP6C (HsPP6C [Hs], NP_002712.1), Caenorhabditis elegans PPh6 (CePPh6 [Ce], NP_497714.2), Drosophila melanoqaster PPV (DmPPV [Dm], NP_511061.1), Saccharomyces cerevisiae SIT4 (ScSIT4 [Sc], NP_010236.1), Schizosaccharomyces pombe Ppe1 (SpPpe1 [Sp], NP_588420.1). The conserved catalytic motifs and active-site residues are marked with boxed. C. Phylogenetic reconstruction of PP6 orthologs was performed using the neighbor-joining method with 1,000 bootstrap replicates. (TIF) [file ppat.1013318.s001.tif]

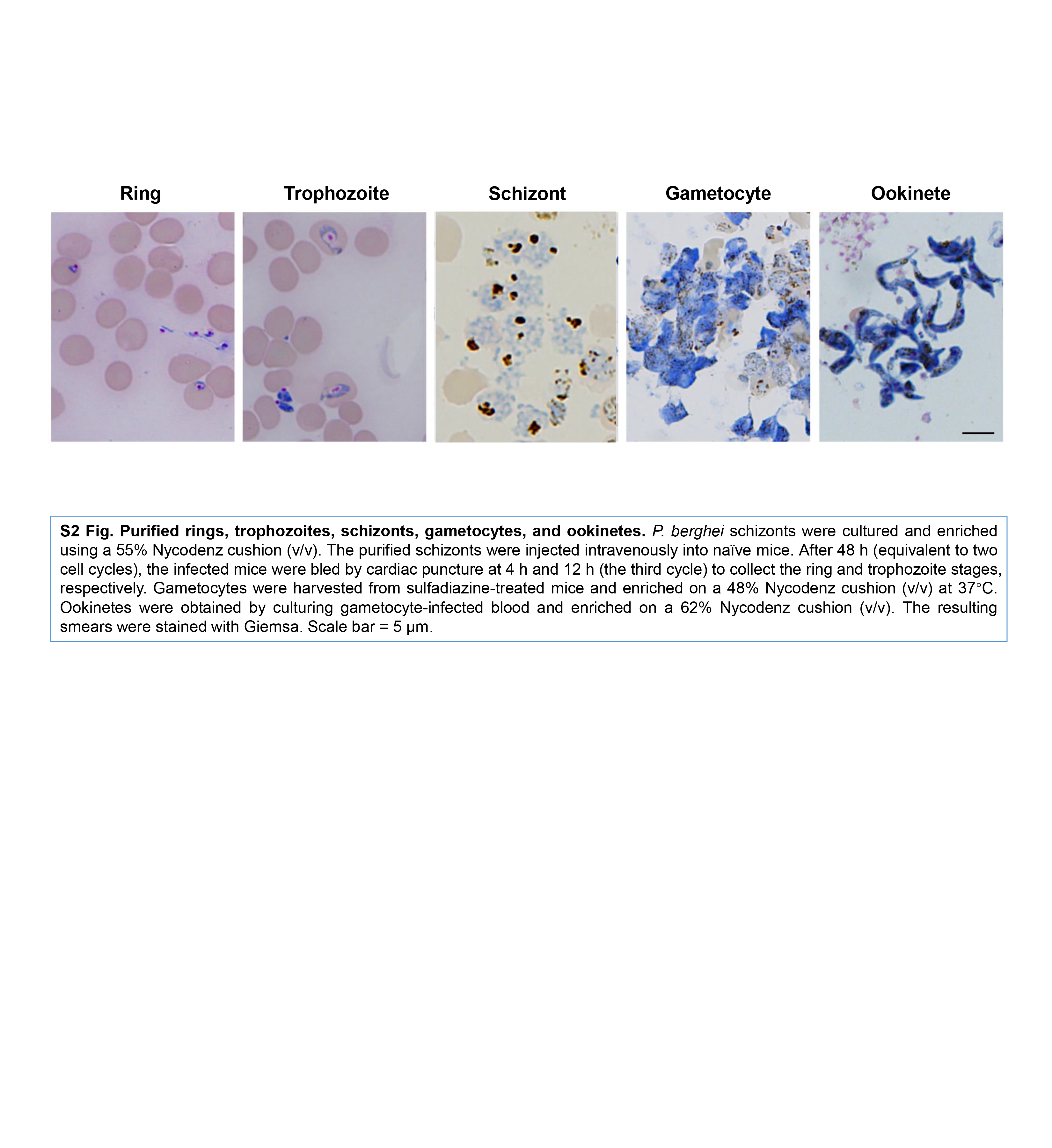

Supplement: S2 Fig — P. berghei schizonts were cultured and enriched using a 55% Nycodenz cushion (v/v). The purified schizonts were injected intravenously into naïve mice. After 48 h (equivalent to two cell cycles), the infected mice were bled by cardiac puncture at 4 h and 12 h (the third cycle) to collect the ring and trophozoite stages, respectively. Gametocytes were harvested from sulfadiazine-treated mice and enriched on a 48% Nycodenz cushion (v/v) at 37°C. Ookinetes were obtained by culturing gametocyte-infected blood and enriched on a 62% Nycodenz cushion (v/v). The resulting smears were stained with Giemsa. Scale bar = 5 µm. (TIF) [file ppat.1013318.s002.tif]

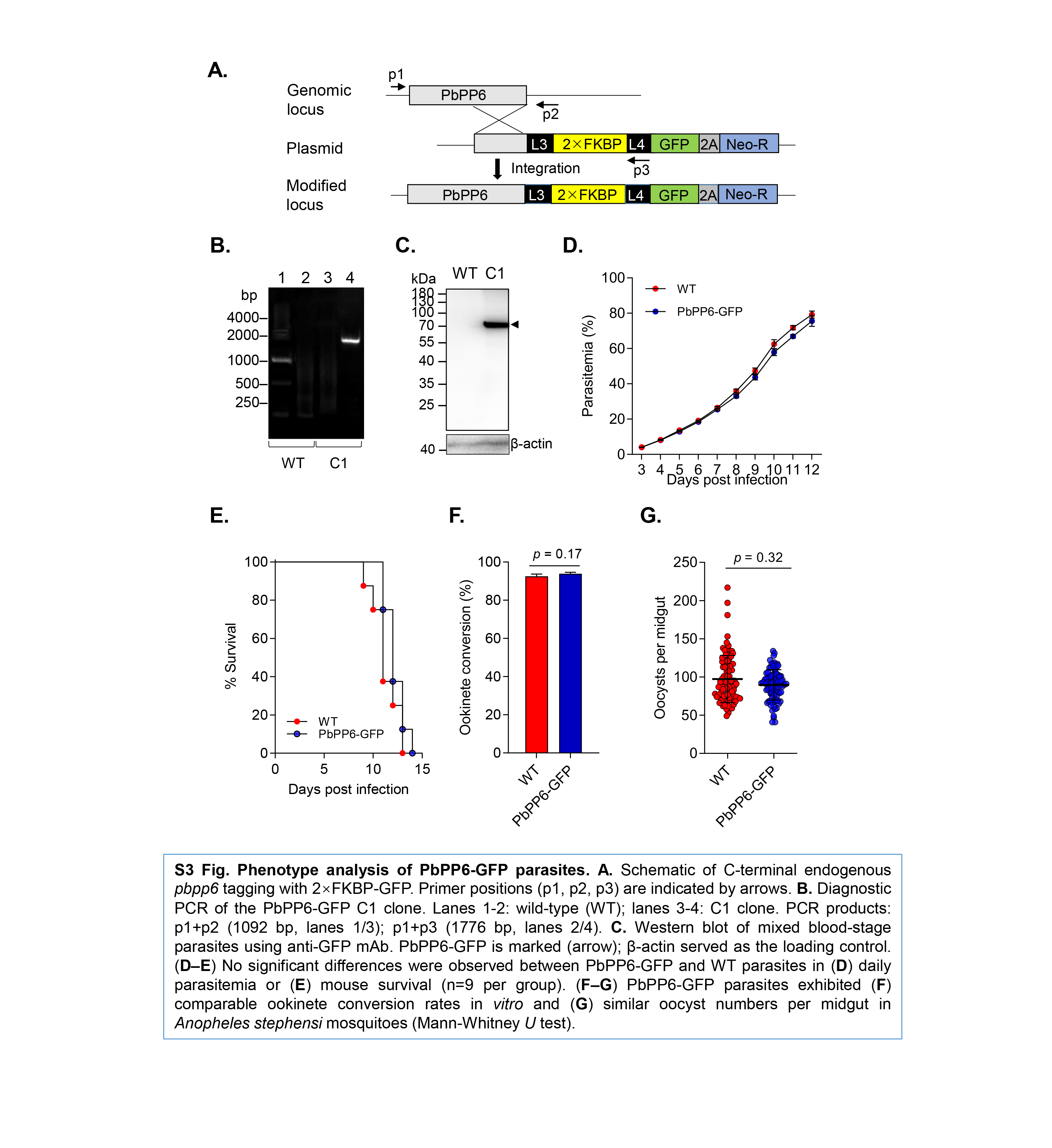

Supplement: S3 Fig — A. Schematic of C-terminal endogenous pbpp6 tagging with 2 × FKBP-GFP. Primer positions (p1, p2, p3) are indicated by arrows. B. Diagnostic PCR of the PbPP6-GFP C1 clone. Lanes 1–2: wild-type (WT); lanes 3–4: C1 clone. PCR products: p1 + p2 (1092 bp, lanes 1/3); p1 + p3 (1776 bp, lanes 2/4). C. Western blot of mixed blood-stage parasites using anti-GFP mAb. PbPP6-GFP is marked (arrow); β-actin served as the loading control. (D–E) No significant differences were observed between PbPP6-GFP and WT parasites in (D) daily parasitemia or (E) mouse survival (n = 9 per group). (F–G) PbPP6-GFP parasites exhibited (F) comparable ookinete conversion rates in vitro and (G) similar oocyst numbers per midgut in Anopheles stephensi mosquitoes (Mann-Whitney U test). (TIF) [file ppat.1013318.s003.tif]

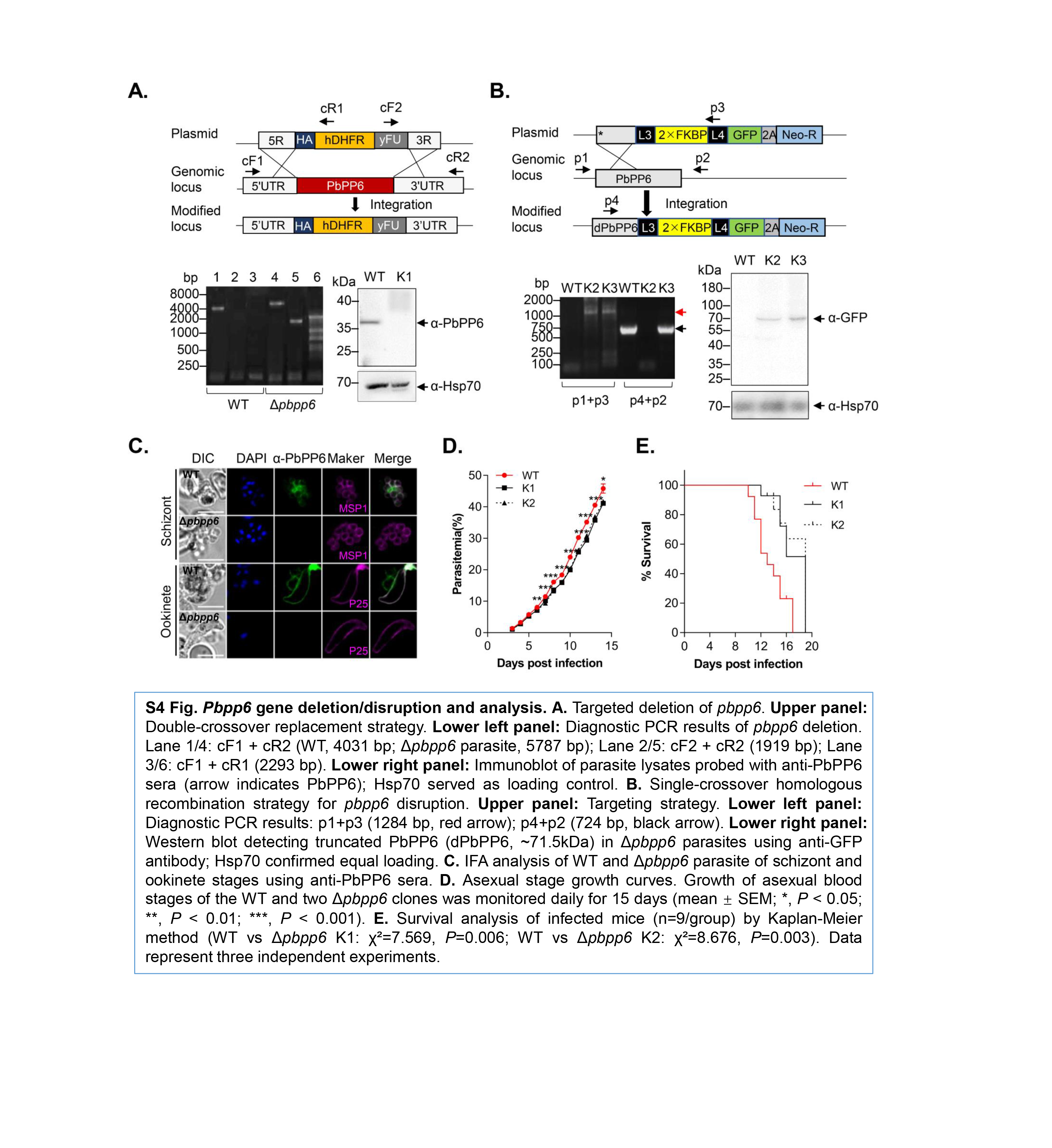

Supplement: S4 Fig — A. Targeted deletion of pbpp6. Upper panel: Double-crossover replacement strategy. Lower left panel: Diagnostic PCR results of pbpp6 deletion. Lane 1/4: cF1 + cR2 (WT, 4031 bp; Δpbpp6 parasite, 5787 bp); Lane 2/5: cF2 + cR2 (1919 bp); Lane 3/6: cF1 + cR1 (2293 bp). Lower right panel: Immunoblot of parasite lysates probed with anti-PbPP6 sera (arrow indicates PbPP6); Hsp70 served as loading control. B. Single-crossover homologous recombination strategy for pbpp6 disruption. Upper panel: Targeting strategy. Lower left panel: Diagnostic PCR results: p1 + p3 (1284 bp, red arrow); p4 + p2 (724 bp, black arrow). Lower right panel: Western blot detecting truncated PbPP6 (dPbPP6, ~ 71.5kDa) in Δpbpp6 parasites using anti-GFP antibody; Hsp70 confirmed equal loading. C. IFA analysis of WT and Δpbpp6 parasite of schizont and ookinete stages using anti-PbPP6 sera. D. Asexual stage growth curves. Growth of asexual blood stages of the WT and two Δpbpp6 clones was monitored daily for 15 days (mean ± SEM; *, P < 0.05; **, P < 0.01; ***, P < 0.001). E. Survival analysis of infected mice (n = 9/group) by Kaplan-Meier method (WT vs Δpbpp6 K1: χ² = 7.569, P = 0.006; WT vs Δpbpp6 K2: χ² = 8.676, P = 0.003). Data represent three independent experiments. (TIF) [file ppat.1013318.s004.tif]

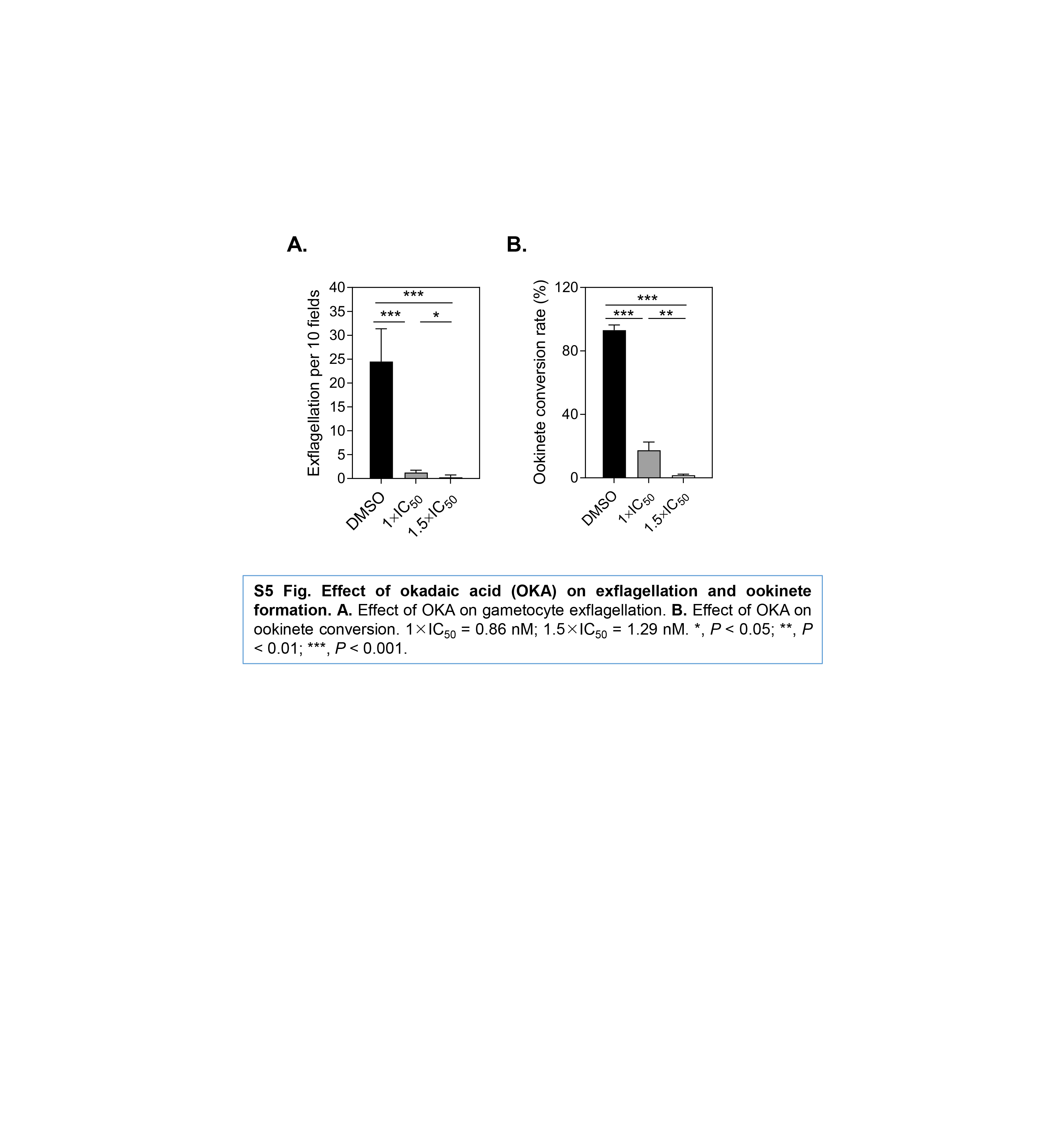

Supplement: S5 Fig — A. Effect of OKA on gametocyte exflagellation. B. Effect of OKA on ookinete conversion. 1 × IC50 = 0.86 nM; 1.5 × IC50 = 1.29 nM. *, P < 0.05; **, P < 0.01; ***, P < 0.001. (TIF) [file ppat.1013318.s005.tif]

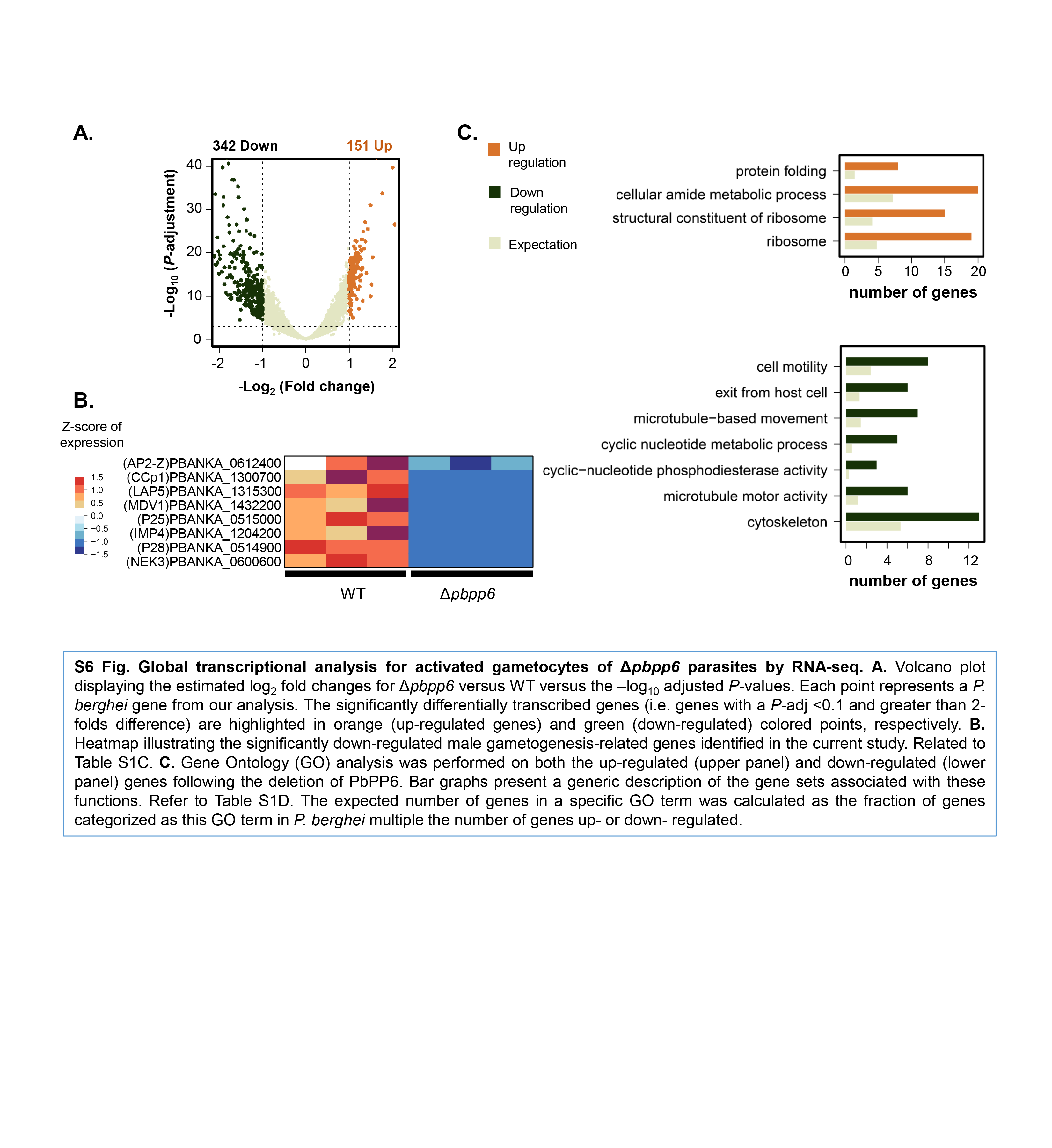

Supplement: S6 Fig — A. Volcano plot displaying the estimated log2 fold changes for Δpbpp6 versus WT versus the –log10 adjusted P-values. Each point represents a P. berghei gene from our analysis. The significantly differentially transcribed genes (i.e., genes with a P-adj < 0.1 and greater than 2-folds difference) are highlighted in orange (up-regulated genes) and green (down-regulated) colored points, respectively. B. Heatmap illustrating the significantly down-regulated male gametogenesis-related genes identified in the current study. Related to S1C Table. C. Gene Ontology (GO) analysis was performed on both the up-regulated (upper panel) and down-regulated (lower panel) genes following the deletion of PbPP6. Bar graphs present a generic description of the gene sets associated with these functions. Refer to S1D Table. The expected number of genes in a specific GO term was calculated as the fraction of genes categorized as this GO term in P. berghei multiple the number of genes up- or down- regulated. (TIF) [file ppat.1013318.s006.tif]

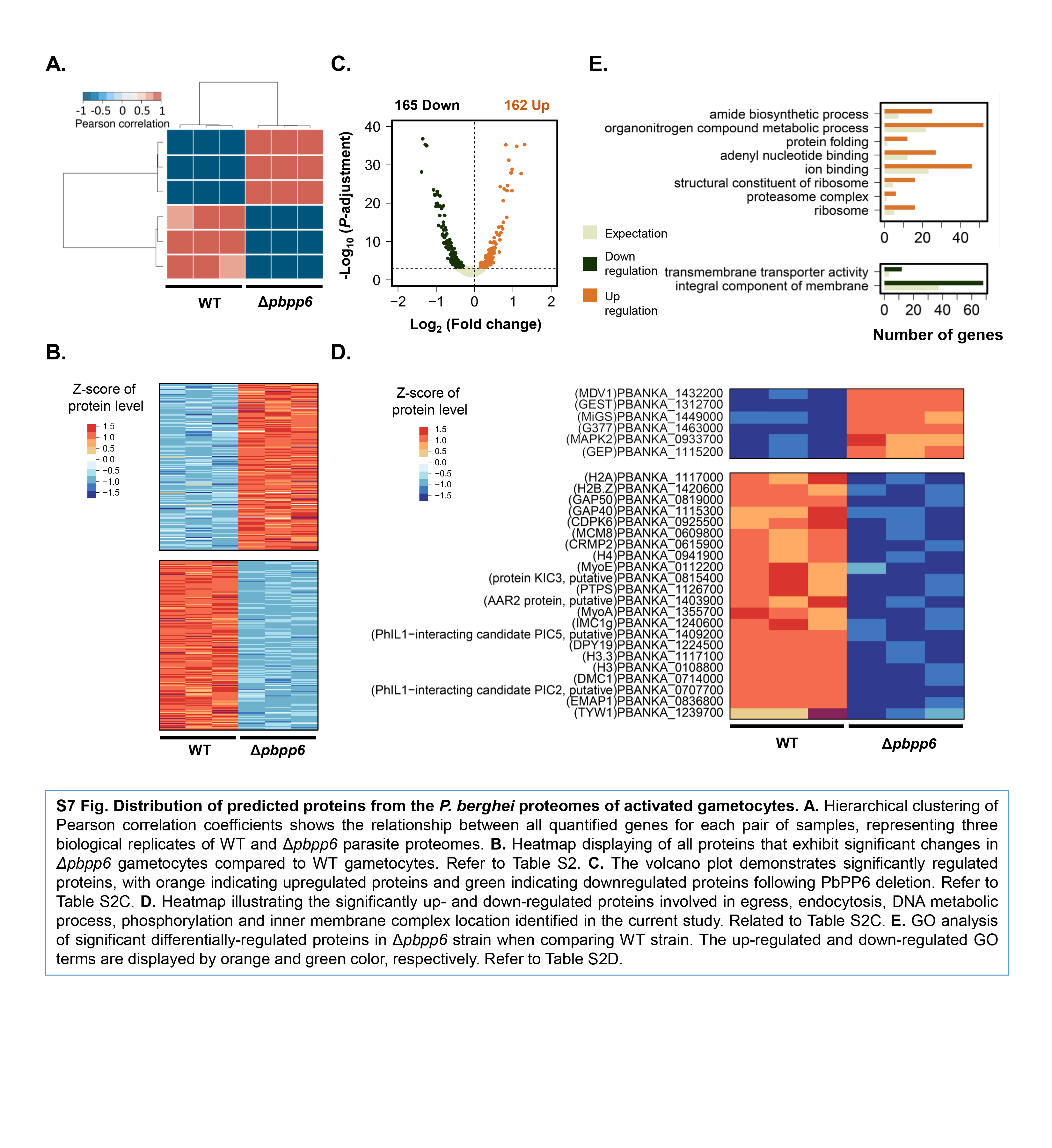

Supplement: S7 Fig — A. Hierarchical clustering of Pearson correlation coefficients shows the relationship between all quantified genes for each pair of samples, representing three biological replicates of WT and Δpbpp6 parasite proteomes. B. Heatmap displaying of all proteins that exhibit significant changes in Δpbpp6 gametocytes compared to WT gametocytes. Refer to S2 Table. C. The volcano plot demonstrates significantly regulated proteins, with orange indicating upregulated proteins and green indicating downregulated proteins following PbPP6 deletion. Refer to S2C Table. D. Heatmap illustrating the significantly up- and down-regulated proteins involved in egress, endocytosis, DNA metabolic process, phosphorylation and inner membrane complex location identified in the current study. Related to S2C Table. E. GO analysis of significant differentially-regulated proteins in Δpbpp6 strain when comparing WT strain. The up-regulated and down-regulated GO terms are displayed by orange and green color, respectively. Refer to S2D Table. (TIF) [file ppat.1013318.s007.tif]

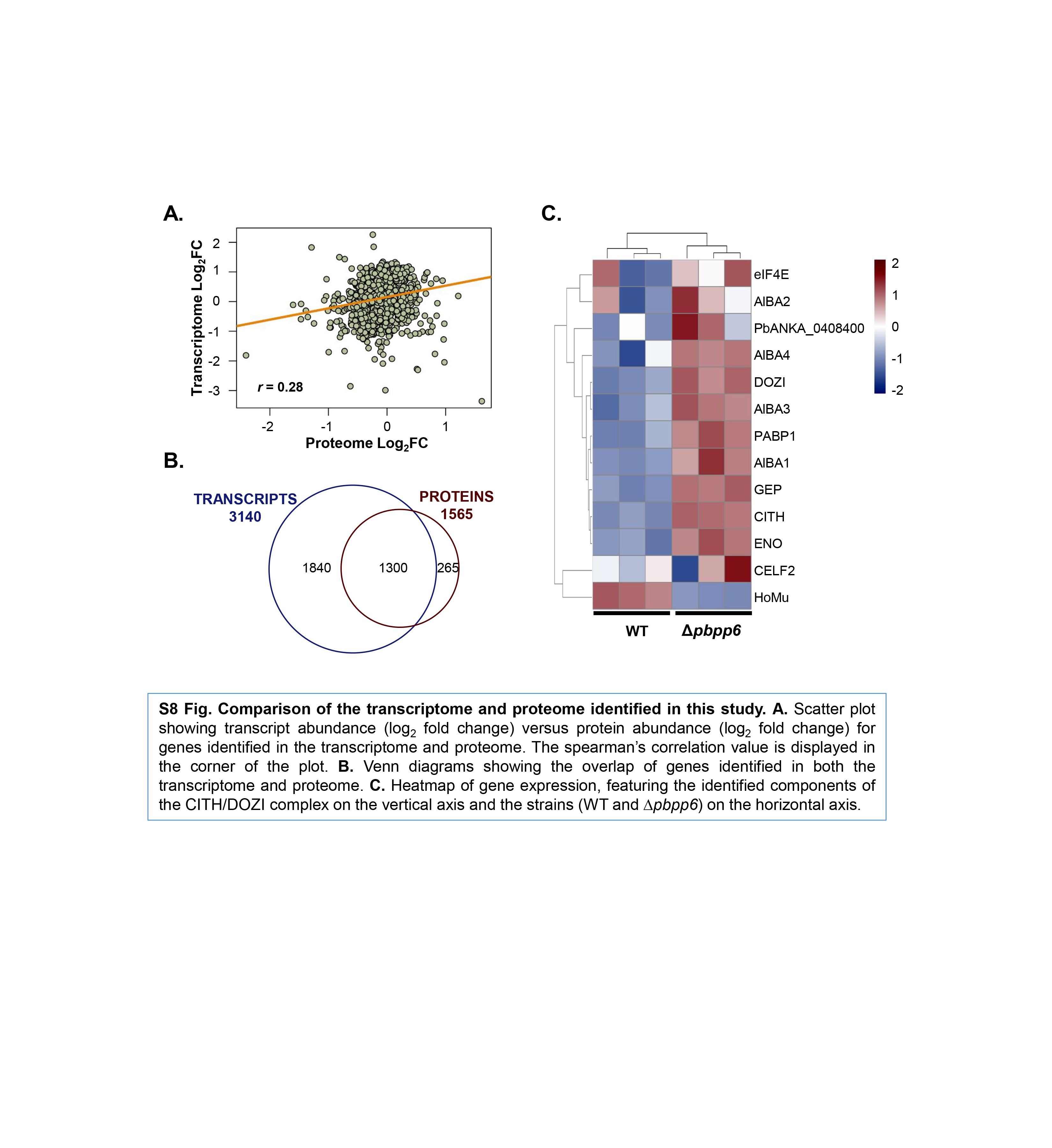

Supplement: S8 Fig — A. Scatter plot showing transcript abundance (log2 fold change) versus protein abundance (log2 fold change) for genes identified in the transcriptome and proteome. The spearman’s correlation value is displayed in the corner of the plot. B. Venn diagrams showing the overlap of genes identified in both the transcriptome and proteome. C. Heatmap of gene expression, featuring the identified components of the CITH/DOZI complex on the vertical axis and the strains (WT and ∆pbpp6) on the horizontal axis. (TIF) [file ppat.1013318.s008.tif]

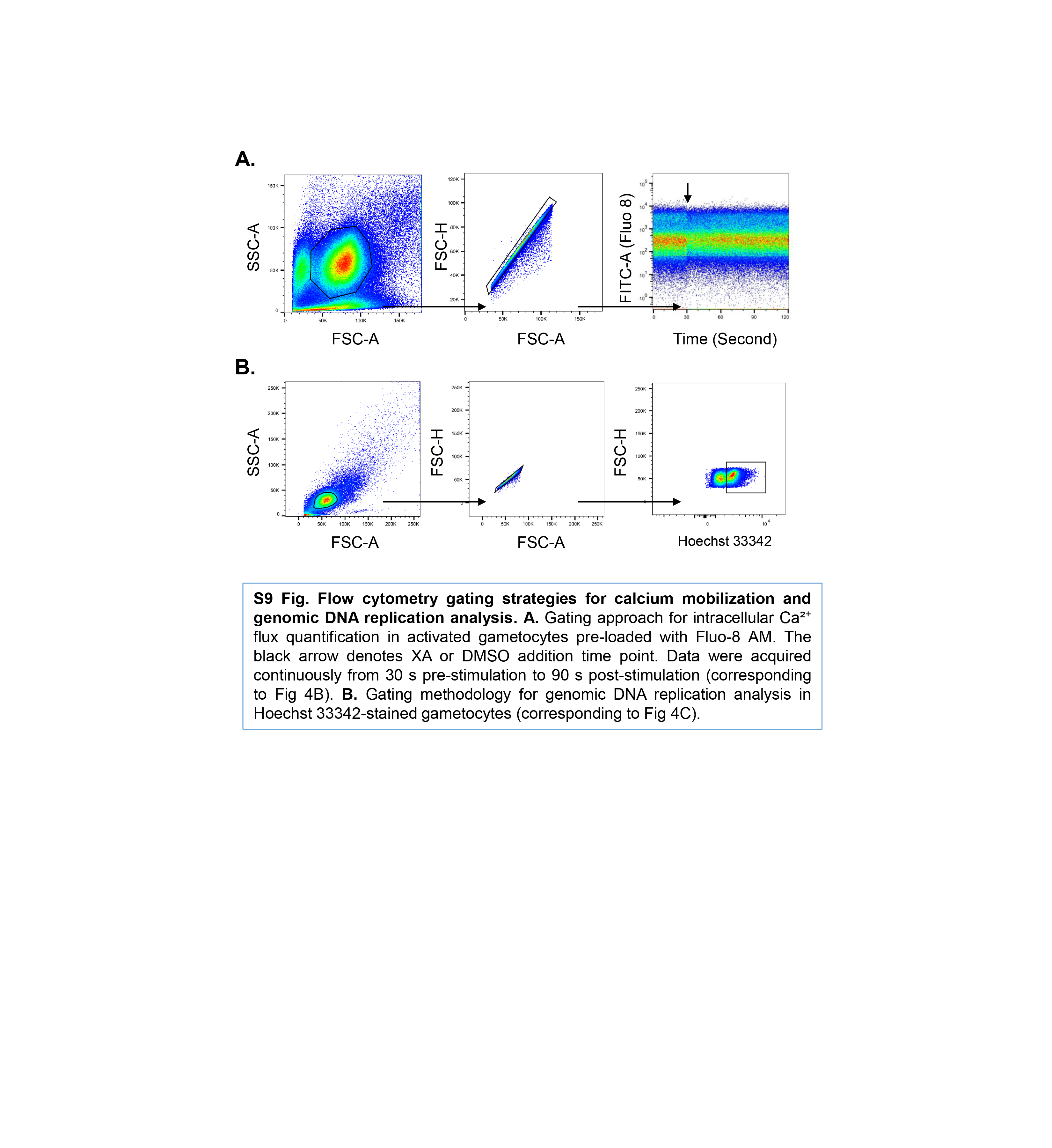

Supplement: S9 Fig — A. Gating approach for intracellular Ca2⁺ flux quantification in activated gametocytes pre-loaded with Fluo-8 AM. The black arrow denotes XA or DMSO addition time point. Data were acquired continuously from 30 s pre-stimulation to 90 s post-stimulation (corresponding to Fig 4B). B. Gating methodology for genomic DNA replication analysis in Hoechst 33342-stained gametocytes (corresponding to Fig 4C). (TIF) [file ppat.1013318.s009.tif]
